# Astrocyte-derived hepcidin controls iron traffic at the blood-brain-barrier via regulating ferroportin 1 of microvascular endothelial cells

**DOI:** 10.1038/s41419-022-05043-w

**Published:** 2022-08-01

**Authors:** Linhao You, Pan-Pan Yu, Tianyu Dong, Wenhuan Guo, Shiyang Chang, Bingjie Zheng, Yunzhe Ci, Fudi Wang, Peng Yu, Guofen Gao, Yan-Zhong Chang

**Affiliations:** 1 Laboratory of Molecular Iron Metabolism, Ministry of Education Key Laboratory of Molecular and Cellular Biology, Hebei Key Laboratory of Animal Physiology, Biochemistry and Molecular Biology, College of Life Sciences, Hebei Normal University, 050024 Shijiazhuang, Hebei Province China; 2 Department of Nutrition, Research Center for Nutrition and Health, Institute of Nutrition and Food Safety, School of Public Health, School of Medicine, Zhejiang University, Hangzhou, 310058 Zhejiang Province China

**Keywords:** Blood-brain barrier, Neurological disorders

## Abstract

Brain iron dysregulation associated with aging is closely related to motor and cognitive impairments in neurodegenerative diseases. The regulation of iron traffic at the blood-brain barrier (BBB) is crucial to maintain brain iron homeostasis. However, the specific mechanism has not been clarified in detail. Using various conditional gene knockout and overexpression mice, as well as cell co-culture of astrocyte and bEND.3 in the transwell, we found that astrocyte hepcidin knockdown increased the expression of ferroportin 1 (FPN1) of brain microvascular endothelial cells (BMVECs), and that it also induced brain iron overload and cognitive decline in mice. Moreover, BMVECs FPN1 knockout decreased iron contents in the cortex and hippocampus. Furthermore, hepcidin regulates the level of FPN1 of BMVECs with conditional gene overexpression in vivo and in vitro. Our results revealed that astrocytes responded to the intracellular high iron level and increased the secretion of hepcidin, which in turn diminished iron uptake at BBB from circulation through directly regulating FPN1 of BMVECs. Our results demonstrate that FPN1 of BMVECs is a gateway for iron transport into the brain from circulation, and the controller of this gateway is hepcidin secreted by astrocyte at its endfeet through physical contact with BMVECs. This regulation is indeed the major checkpoint for iron transport from the blood circulation to the brain. This study delineates the pathway and regulation of iron entry into the brain, providing potential therapeutic targets for iron dysregulation-related neurological diseases.

## Introduction

As populations age, an urgent need has emerged to prevent and treat neurological diseases related to aging. Brain iron dysregulation associated with aging is closely related to motor and cognitive impairments [1]. Accumulating evidence shows that iron deficiency and anemia are associated with impaired neurocognitive development in young children [2, 3], whereas iron overload is involved in the pathogenesis of neurodegenerative diseases, including Alzheimer’s disease (AD) and Parkinson’s disease (PD), by activating apoptosis or ferroptosis [4–6]. Therefore, brain iron metabolism must be tightly regulated to maintain the normal physiological function of the brain. The specific mechanisms that regulate iron metabolism in the brain are not well understood.

Iron homeostasis in the brain is controlled by brain iron uptake and release, including iron transport across the blood-brain-barrier (BBB), which is the main site controlling brain iron level [7]. The available data suggested that the transferrin (Tf)/transferrin receptor (TfR1) pathway may be the major route for iron to enter brain tissue through the luminal membrane of capillary endothelial cells [3, 8–10], whereas the ferroportin 1 (FPN1) on the abluminal side of endothelial cells may be the crucial molecule for iron release into the brain tissue [11–13]. However, whether the FPN1 on the membrane of brain microvascular endothelial cells (BMVECs) plays a key role in the transport of iron into the brain and how its level is regulated have not been fully understood.

Hepcidin, an iron-regulatory hormone, plays an essential role in maintaining systemic iron homeostasis [2, 14, 15]. In the brain, hepcidin immunoreactivity can be detected in both neurons and GFAP-positive glial cells in various brain regions [16]. Several studies have indicated an important regulatory role for hepcidin in the brain [17, 18]. *Hamp* mRNA levels in different brain regions increased with aging [12], and injection of hepcidin into the lateral cerebral ventricle decreased iron influx into brain tissue and attenuated brain iron overload [19–21]. Hepcidin could also reduce the expression of inflammatory factors in astrocytes and microglia treated by β-amyloid (Aβ) aggregates [22]. These findings suggest that hepcidin might be an important regulator of brain iron homeostasis, and a potentially valuable target for the treatment of brain tissue iron overload, such as seen in AD. In vitro studies have shown that hepcidin down-regulated iron efflux from BMVECs by inducing FPN1 internalization [11, 23]. However, it remained uncertain whether hepcidin is the major regulator of iron traffic at BBB in vivo, and whether this regulatory mechanism effectively controls brain iron uptake.

In this study, using several conditional gene knockout or overexpression mouse models, in combination with in vitro experiments, we demonstrated the key roles of astrocyte hepcidin and its interfacial physical interaction with BMVECs’ FPN1 in the regulation of brain iron uptake at BBB, and that astrocytes responded to the changes intracellular iron and thereby regulated total iron level in the brain. This study provided potential therapeutic targets for iron dysregulation-related neurological diseases.

## Results

### Astrocyte hepcidin knockdown induced brain iron overload and cognitive decline whereas FPN1 increased in BMVECs

**Fig. 1:**
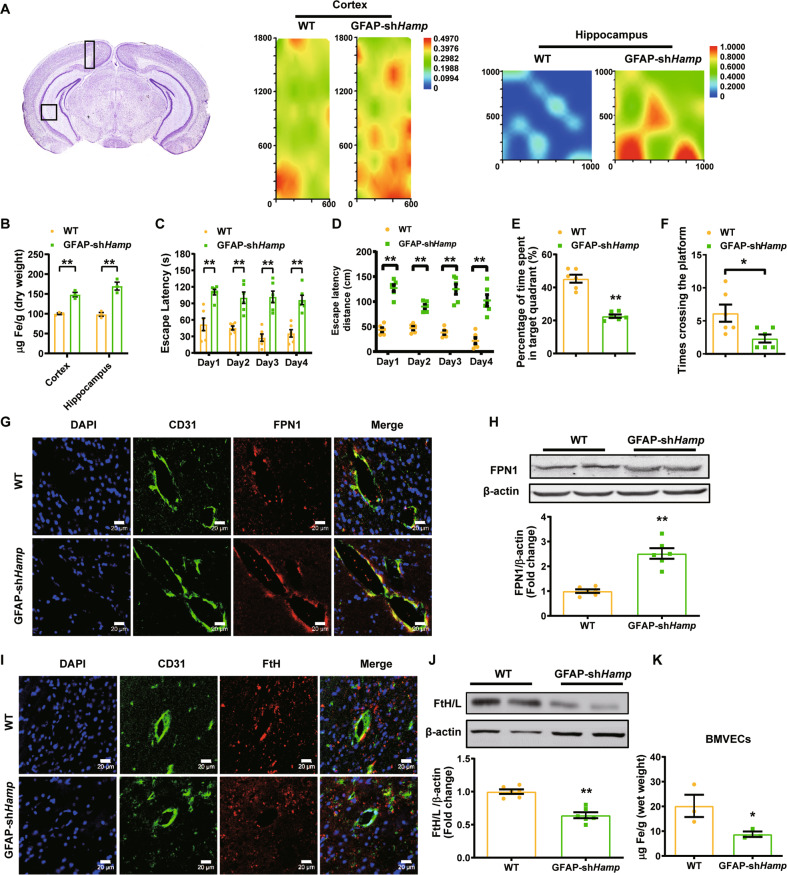
Astrocyte hepcidin knockdown induced brain iron overload and cognitive decline whereas FPN1 increased in BMVECs. **A** is iron content in the cortex and hippocampus of 12-month-old GFAP-sh*Hamp* and WT mice measured by SR-XRF. Rectangles in the brain sections in the left panels indicate the areas scanned showing in the right-hand panels. **B** is total iron in the cortex and hippocampus of GFAP-sh*Hamp* and WT mice determined by ICP-MS. Data are presented as the mean ± SEM, *n* = 3, **p < 0.01 GFAP-sh*Hamp* vs. WT group. **C-F** were GFAP-sh*Hamp* and WT mice subjected to a water maze test. The escape latency of time (**C**), path length (**D**), times of crossing the platform after removing the platform at the fifth day (**E**) and percentage of time spent in target quadrant (**F**) are presented as the mean ± SEM, *n* = 6, *p < 0.05 and **p < 0.01. **G** is immunofluorescent staining of CD31 (endothelial cell marker) and FPN1 in the brain of WT and GFAP-sh*Hamp* mice. Scale bar = 20 μm. **H** is the level of FPN1 protein in BMVECs isolated from WT and GFAP-sh*Hamp* mice detected by western blot analysis. The relative expression levels were calculated after they were normalized to β-actin levels, and expressed as the mean ± SEM, *n* = 4 (WT) vs. *n* = 6 (GFAP-sh*Hamp*), **p < 0.01. **I** is immunofluorescent staining of CD31 and FtH in the brain in WT and GFAP-sh*Hamp* mice. Scale bar = 20 μm. **J** is the level of ferritin protein in BMVECs. The relative expression levels were calculated after they were normalized to β-actin levels and expressed as the mean ± SEM, *n* = 4 (WT) vs. *n* = 6 (GFAP-sh*Hamp*), **p < 0.01 vs. WT group. **K** is iron in BMVECs isolated from WT and GFAP-sh*Hamp* mice detected by ICP-MS. Data are presented as the mean ± SEM, *n* = 3, *p < 0.05 vs. WT group.

To investigate if the astrocyte hepcidin play a critical regulatory role in brain iron uptake, we constructed two mouse models, astrocyte hepcidin preferential knockdown mice (GFAP-sh*Hamp*) and overexpression mice (*Hamp*^*Gfap*^ OE). The differences in hepcidin expression in astrocytes of these mice were confirmed by immunofluorescent staining (Figure S1A). We detected the total iron levels in GFAP-sh*Hamp* and wild-type (WT) mice by both synchrotron radiation X-ray fluorescence and ICP-MS. The total iron level in GFAP-sh*Hamp* mice was found to be significantly increased in the cortex and hippocampus compared with the WT control mice (Fig. 1A and B). The Morris water maze test showed that, compared with WT mice, GFAP-sh*Hamp* mice had impaired cognitive function, as shown by the significantly longer time and distance to reach the platform (Fig. 1C and D). In addition, after removing the platform, the GFAP-sh*Hamp* mice showed lower times crossing over the platform and shorter time spent in the target quadrant than the WT mice (Fig. 1E and F). Consistent with that, the GFAP-sh*Hamp* mice had an increased Aβ expression and a decreased Bcl2/Bax ratio (Figure S1B and C). Overall, these results suggested that the GFAP-sh*Hamp* mice had brain iron overload accompanied by cognitive impairment, indicating the important role of astrocyte hepcidin in regulating brain iron homeostasis.

To examine whether the astrocyte hepcidin affects the expression of FPN1 on the membrane of BMVECs, we determined the FPN1 expression in BMVECs by immunofluorescence staining and by western blot analysis of the isolated BMVECs from mice. The GFAP-sh*Hamp* mice had increased FPN1 protein expression in BMVECs (Fig. 1G and H), and a decrease in iron storage protein ferritin-H (FtH) (Fig. 1I) and FtH/ferritin-L (FtL) (Fig. 1J) in endothelial cells. These indicated that the knockdown of astrocyte hepcidin enhanced FPN1 level on the membrane of BMVECs, which then released more iron into the brain tissue, resulting in an iron-deficient status in the endothelial cells. The iron deficiency of endothelial cells was also confirmed by ICP-MS analysis of iron concentration (Fig. 1K). The opposite effect was observed in *Hamp*^*Gfap*^ OE mice which showed reduced FPN1 protein expression and raised ferritin level in endothelial cells, and decreased iron level in the cortex (Figure S1D–F). These observations imply that astrocyte hepcidin is a critical molecule in the regulation of brain iron homeostasis, and it may regulate brain iron influx by controlling the level of FPN1 on the membrane of BMVECs.

### Hepcidin regulates the level of FPN1 of BMVECs

**Fig. 2:**
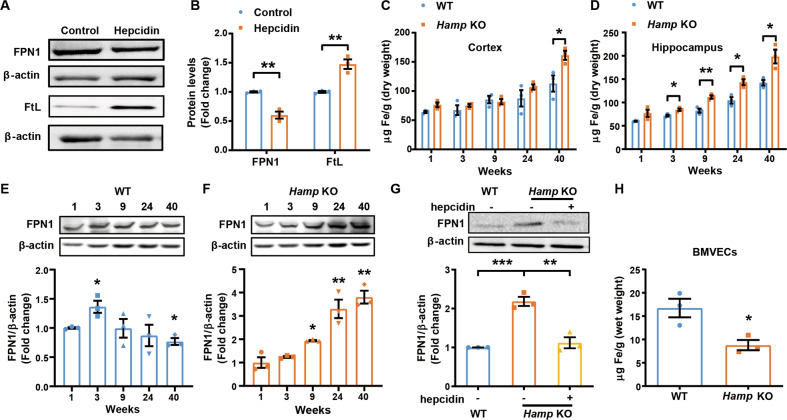
Hepcidin regulates the levels of FPN1 of BMVECs. **A** is western blot analysis of FPN1 and ferritin levels in BMVECs 24 h following ICV injection of hepcidin into 3-month-old WT mice. **B** is the relative expression levels of FPN1 and ferritin in control and hepcidin-treated mice calculated after they were normalized to β-actin levels and expressed as the mean ± SEM. *n* = 3, **p < 0.01. **C**, **D** are the total iron in the cerebral cortex (**C**), hippocampus (**D**) of *Hamp* KO or control WT mice of the indicated ages determined by ICP-MS. Values are presented as the mean ± SEM, *n* = 3, *p < 0.05 and **p < 0.01. **E** is the FPN1 protein levels in BMVECs of WT mice of the indicated ages. The relative expression levels were expressed as the mean ± SEM, *n* = 3, *p < 0.05 vs. 1-week-old mice. **F** is the FPN1 protein levels in BMVECs of *Hamp* KO mice of the indicated ages. The relative expression levels were expressed as the mean ± SEM, *n* = 3, *p < 0.05 and **p < 0.01 vs. 1-week-old mice. **G** is the protein levels of FPN1 in BMVECs measured by western blot analysis following ICV injection of hepcidin in 24-week-old *Hamp* KO and WT mice. The relative expression levels were normalized and expressed as the mean ± SEM, *n* = 3, **p < 0.01 and ***p < 0.001. **H** is total iron level in BMVECs determined by ICP-MS in 24-week-old *Hamp* KO and WT mice. The values are presented as the mean ± SEM, *n* = 3, *p < 0.05 vs. WT group.

To determine whether hepcidin peptide regulates FPN1 of BMVECs in the brain, we detected the levels of FPN1 protein from the isolated BMVECs of the cortex and hippocampus of WT mice following intracerebral-ventricular (ICV) injection of hepcidin. FPN1 of BMVECs decreased in response to hepcidin administration, accompanied by the expected increase in ferritin concentration in BMVECs (Fig. 2A and B). To further confirm the role of endogenous hepcidin in brain iron homeostasis, we utilized global hepcidin knockout mice (*Hamp* KO) to investigate the effect of hepcidin on FPN1 of BMVECs. In *Hamp* KO mice, brain iron levels gradually increased with age up to at least 40-week old in the cerebral cortex and hippocampus (Fig. 2C and D). A similar trend was observed in WT mice, as seen in previous studies [6, 24], but this phenomenon was magnified in the *Hamp* KO mice. Notably, in the hippocampus of *Hamp* KO mice, the increase of iron was significant as early as in 3-week-old mice compared with the control mice (Fig. 2D). In the BMVECs of WT mice, FPN1 protein levels peaked at the age of 3 weeks old, but declined thereafter (Fig. 2E). This may be due to the gradual increase in hepcidin expression with aging [12]. However, in the absence of hepcidin, FPN1 protein expression was the lowest in one-week-old mice, and increased gradually with age in BMVECs up to 40 weeks (Fig. 2F). ICV administration of human hepcidin into 24-week-old *Hamp* KO mice led to a decrease in FPN1 protein in BMVECs approximately to that of the aged-matched control mice (Fig. 2G). In addition, we found that the iron levels in BMVECs were significantly decreased in *Hamp* KO mice (Fig. 2H). These findings indicate that the level of FPN1 protein in BMVECs is modulated by hepcidin levels in the brain.

### The FPN1 of BMVECs is a key molecule for brain iron uptake

**Fig. 3:**
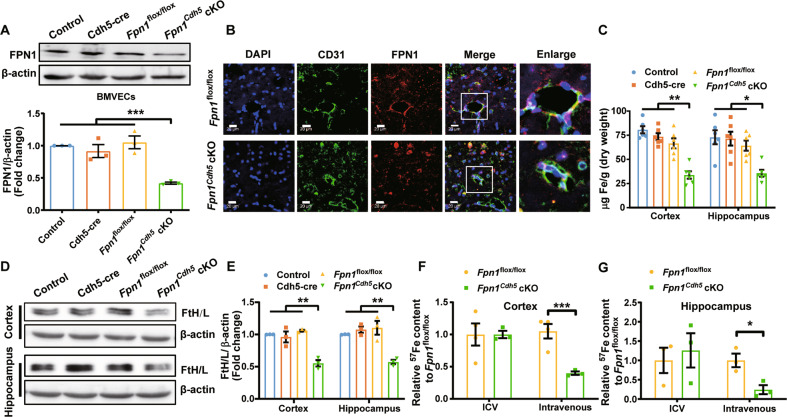
FPN1 of BMVECs is a key molecule for brain iron uptake. **A** is western blot analysis of FPN1 protein levels in BMVECs of 9-week-old *Fpn1*^Cdh5^ cKO and different control mice. The relative expression levels were normalized to β-actin levels and presented as the mean ± SEM, *n* = 3, ***p < 0.001. **B** is immunofluorescent staining of CD31 and FPN1 in hippocampus of *Fpn1*^Cdh5^ cKO and control mice. Higher magnification images are shown in the last column. Scale bar = 20 μm. **C** is total brain iron determined by ICP-MS in 9-week-old *Fpn1*^Cdh5^ cKO and control mice. The values are presented as the mean ± SEM, *n* = 6, *p < 0.05 and **p < 0.01. **D** is ferritin protein level in the cortex and hippocampus of *Fpn1*^Cdh5^ cKO and control mice. **E** is the relative expression levels normalized to β-actin levels and expressed as the mean ± SEM, *n* = 3, **p < 0.01. **F**, **G** are the levels of ^57^Fe in cortex (**F**) and hippocampus (**G**) of 3-month-old *Fpn1*^Cdh5^ cKO and *Fpn1*^flox/flox^ control mice determined by ICP-MS following intravenous injection of ^57^Fe. Values are presented as the mean ± SEM, *n* = 4 (*Fpn1*^flox/flox^) vs. *n* = 3 (*Fpn1*^Cdh5^ cKO), *p < 0.05 and ***p < 0.001.

To assess whether FPN1 in BMVECs plays a critical role in iron transport across the endothelial cells into the brain, we crossed *Fpn1*^flox/flox^ mice with VE-Cadherin (Cdh5)-Cre mice to generate conditional knockout mice ablating of FPN1 in capillary endothelial cells (*Fpn1*^*Cdh5*^ cKO). The genotype of the mice was verified by primer-specific PCR (Figure S2A and B). We extracted BMVECs from the *Fpn1*^*Cdh5*^ cKO mice and detected the expression of FPN1. As determined by western blot analysis, the expression of FPN1 protein in BMVECs was substantially decreased (Fig. 3A). By immunofluorescence, the expression of FPN1 in BMVECs was almost undetectable in the hippocampus of the *Fpn1*^*Cdh5*^ cKO mice (Fig. 3B), indicating effective FPN1 knockout in BMVECs.

To investigate the effect of FPN1 depletion in BMVECs on brain iron level, we determined the total iron levels by ICP-MS and the ferritin protein levels in the cortex and hippocampus by western blot. In the *Fpn1*^*Cdh5*^ cKO mice, the total iron levels were decreased significantly (Fig. 3C and S2C), and the FtH and FtL protein levels were decreased as well (Fig. 3D and E), in comparison to those in the control mice. In contrast, in isolated BMVECs, the total iron and ferritin levels were elevated in *Fpn1*^*Cdh5*^ cKO mice (Figure S2D and E).

To determine whether the reduced brain iron is due to a low uptake of iron into the brain or a high iron release from the brain, we measured radio-iron in the brain following ICV or intravenous injection of ^57^FeCl_3_ into *Fpn1*^*Cdh5*^ cKO and control mice. Compared to controls, the levels of ^57^Fe in the cortex and hippocampus of *Fpn1*^*Cdh5*^ cKO mice following intravenous injection of ^57^Fe were significant lower than those in the control mice, whereas following ICV injection of ^57^Fe there was no difference between the two groups of mice (Fig. 3F and G). This indicates that iron cannot be efficiently transported from the BMVECs to the brain tissue without FPN1 on abluminal membrane of BMVECs.

### Hepcidin secreted by the endfeet of astrocytes regulates FPN1 of BMVECs via interfacial physical contact

**Fig. 4:**
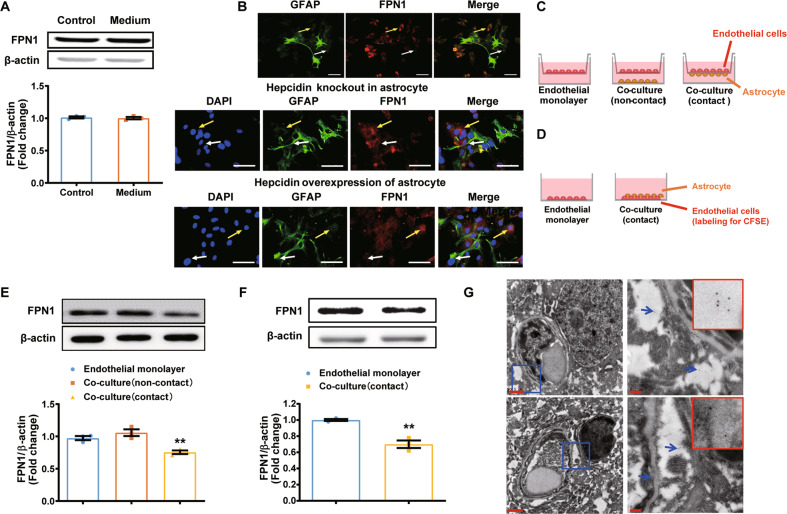
Hepcidin secreted by the endfeet of astrocytes regulates FPN1 on BMVECs via interfacial physical contact in vitro. **A** is the protein levels of FPN1 in bEND.3 cells were detected after incubation in medium conditioned by cultured primary astrocytes, *n* = 3. **B** are bEND.3 cells that co-cultured with control primary astrocytes, primary astrocytes with hepcidin knockdown or primary astrocytes with hepcidin overexpression. bEND.3 cells not contacting astrocytes are indicated with a yellow arrow; cells in contact with astrocytes are indicated with a white arrow. Scale bar = 50 μm. **C** is the schematic representation of the in vitro co-culture test. A bEnd.3 monolayer was grown on a transwell insert with astrocytes grown on the opposite side of the transwell or on the bottom of the culture dish. **D** is the schematic representation of the in vitro co-culture test. bEnd.3 cells were labeled with CFSE, and primary astrocytes were grown on the bottom of the culture dish. **E**, **F** are western blot analysis of FPN1 levels in groups treated as in **C** and **D**. The relative expression levels were normalized to β-actin levels and expressed as the mean ± SEM, *n* = 3, **p < 0.01. **G** The locations of hepcidin were determined by immuno-EM in cortex.

To determine how astrocyte hepcidin regulates the FPN1 of BMVECs, we examined the expression of FPN1 in the mouse BMVEC cell line, bEND.3, after incubating the cells in medium conditioned by cultured primary WT astrocytes. Unexpectedly, we found that the expression of FPN1 in bEND.3 cells was not affected by the medium conditioned by astrocytes (Fig. 4A). Given that the astrocytes are a major component of the BBB structure, we speculated that a cell to cell direct contact between BMVECs and astrocytes may be needed to modulate the transport of iron into the brain. To test this hypothesis, we examined whether hepcidin secreted through the endfeet of astrocytes could affect FPN1 expression in endothelial cells by co-culturing primary mouse astrocytes with bEND.3 cells. We found that the expression of FPN1 in bEND.3 cells was reduced only when there were direct physical contacts between bEND.3 cells and astrocytes (white arrow), but not in the area where there was no direct contact (yellow arrow) (Fig. 4B, upper panel). In addition, we used primary astrocytes isolated from mice lacking hepcidin in co-culture experiments, no change in the expression of FPN1 in bEND.3 cells was observed (Fig. 4B, middle panel); whereas primary astrocytes overexpressing hepcidin caused a significant decrease in FPN1 expression in co-cultured bEND.3 cells, irrespective of whether they were in contact (white arrow) or not (yellow arrow) (Fig. 4B, lower panel). However, the level of FPN1 in bEND.3 cells physically contacted with astrocytes was much lower than that of cells without direct contact. In another transwell co-culture system (Fig. 4C and D), the expression of FPN1 in endothelial cells decreased significantly when the astrocytes and endothelial cells directly contacted (Fig. 4E and F). In addition, by immuno-EM, hepcidin-positive granules were found in the place where the endothelial cells were wrapped by the endfeet of astrocytes (Fig. 4G). These results are consistent with our previous observation that astrocytes and BMVECs need to be in direct contact with each other for astrocyte hepcidin to regulate FPN1 of BMVECs. Taken together, our data indicate that hepcidin secreted by the astrocytes can regulate FPN1 level in endothelial cells only through direct contact.

### Changes in intracellular iron concentration of astrocytes control hepcidin release and consequently regulate brain iron uptake

As observed above, the astrocyte hepcidin played an important role in regulating brain iron uptake, therefore we explored whether astrocytes could sense the changes of iron content in the brain and responsively modulate the expression of hepcidin. The levels of *Hamp* mRNA in astrocytes, microglia and neurons, isolated from adult WT mice by FACS, were determined. We found that the level of *Hamp* mRNA in astrocytes was higher than that in other type of cells (Figure S3). More importantly, we found that astrocyte *Hamp* mRNA increased significantly in response to the changes of brain iron level after ICV injection of FAC (Figure S3), but no significant change of *Hamp* expression was observed in other cells.

**Fig. 5:**
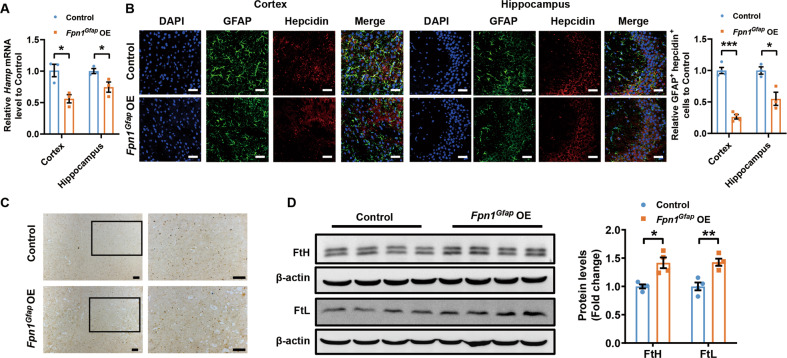
Overexpression of FPN1 in astrocytes decreased the secretion of astrocyte hepcidin and elevated brain iron. **A** is the expression of *Hamp* mRNA detected by qPCR in cortex and hippocampus of 3-month-old control and *Fpn1*^*Gfap*^ OE mice. Values are presented as the mean ± SEM, *n* = 3, *p < 0.05. **B** is double immunofluorescence labelling of hepcidin (red) and GFAP (green) carried out in control and *Fpn1*^*Gfap*^ OE mice. Scale bar = 40 μm. The numbers of GFAP and hepcidin-positive cells were counted in five separate fields and are presented as a percentage of the total cells in the fields. Values are presented as the mean ± SEM, *n* = 3, *p < 0.05, ***p < 0.001. **C** is the iron level detected by Perl’s iron staining in cortex and hippocampus of control mice and *Fpn1*^*Gfap*^ OE mice. Scale bar = 100 μm. **D** are the protein levels of FtL and FtH in cortex. Expression levels were normalized to β-actin and presented as the mean ± SEM. *n* = 4. *p < 0.05, **p < 0.01 vs. control mice.

To verify that the iron levels in astrocytes affect the expression of hepcidin and subsequently modulate brain iron level, we overexpressed FPN1 in astrocytes and investigated its effects on hepcidin expression. We found that *Fpn1*^*Gfap*^ OE mice showed decreased hepcidin expression compared with that of control mice in both cortex and hippocampus (Fig. 5A and B). Meantime, we found that the iron level in cortex of *Fpn1*^*Gfap*^ OE mice significantly increased observed from Perl’s iron staining (Fig. 5C) and the consistently increased expression of FtL and FtH expression levels (Fig. 5D). These results indicate that astrocytes indeed respond to the accumulation of iron in the brain and raise the expression of hepcidin.

### Downregulation of astrocyte FPN1 mitigates iron accumulation in brain and improves cognitive function

**Fig. 6:**
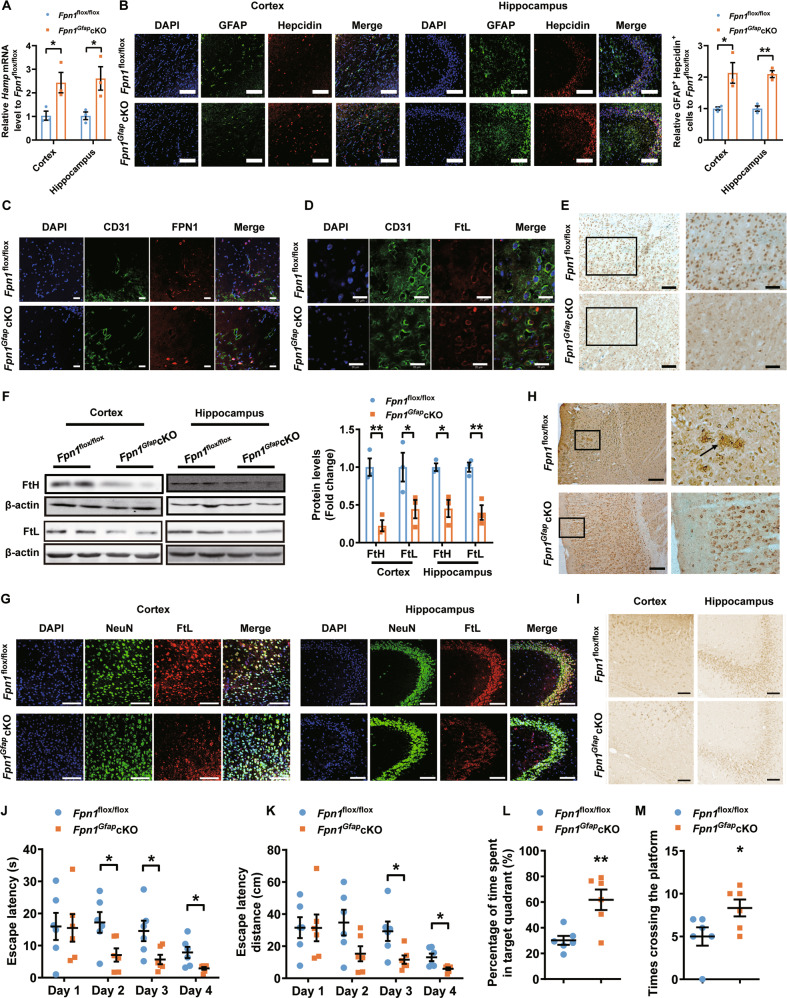
Downregulation of astrocyte FPN1 mitigates iron accumulation in brain and improves cognitive function. **A** is the expression of hepcidin detected by qPCR in cortex and hippocampus of 15-month-old *Fpn1*^flox/flox^ and *Fpn1*^*Gfap*^ cKO mice. Values are presented as the mean ± SEM, *n* = 3, *p < 0.05 vs. *Fpn1*^flox/flox^ mice. **B** is double immunofluorescence labelling of hepcidin (red) and GFAP (green, staining for astrocyte) carried out in 15-month-old *Fpn1*^flox/flox^ and *Fpn1*^*Gfap*^ cKO mice. Scale bar = 100 μm. The number of GFAP and hepcidin-positive cells was counted in five separate fields and are presented as a percentage of the total cells in the fields. Values are presented as the mean ± SEM, *n* = 3, *p < 0.05, **p < 0.01. **C** is double immunofluorescence labelling of CD 31 (green, staining for BMVEC) and FPN1 (red). Scale bar = 20 μm. **D** is double immunofluorescence labelling of CD31 (green) and FtL (red). Scale bar = 20 μm. **E** is the iron level detected by using Perl’s iron staining in cortex and hippocampus of *Fpn1*^flox/flox^ mice and *Fpn1*^*Gfap*^ cKO mice. Scale bar of left panel = 100 μm; scale bar of right panel = 50 μm. **F** are the protein levels of FtL and FtH determined by western blot. Expression levels were normalized to β-actin and presented as the mean ± SEM. *n* = 3. *p < 0.05, ** *p* < 0.01 vs. *Fpn1*^flox/flox^ mice. **G** Double immunofluorescence labelling of FtL (red) and NeuN (green, staining for neurons) was carried out in cortex and hippocampus in 15-month-old *Fpn1*^flox/flox^ and *Fpn1*^*Gfap*^ cKO mice. Scale bar = 100 μm. **H** are the Aβ levels of 15-month-old *Fpn1*^flox/flox^ and *Fpn1*^*Gfap*^ cKO mice detected by immunohistochemistry. Scale bar = 50 μm. **I** are the levels of tau phosphorylation in cortex and hippocampus of *Fpn1*^flox/flox^ and *Fpn1*^*Gfap*^ cKO mice detected by immunohistochemistry. Scale bar = 50 μm. (**J**-**M**) are *Fpn1*^*Gfap*^ cKO and *Fpn1*^flox/flox^ mice subjected to a Morris water maze test. The escape latency of time (**J**), path length (**K**), percentage of time spent in target quadrant (**L**) and times of crossing the platform after removing the platform at the fifth day (**M**) are presented as the mean ± SEM, *n* = 6, *p < 0.05, **p < 0.01.

We crossed the *Fpn1*^flox/flox^ mice with GFAP-Cre mice, generating a mouse model with conditional knockout of FPN1 in astrocyte (*Fpn1*^*Gfap*^ cKO). Genetic identification and immunofluorescence staining results showed that the expression of FPN1 in astrocytes was indeed depleted in *Fpn1*^*Gfap*^ cKO mice (Figure S4), and we did observe the expected increase of iron storage protein level in astrocyte after conditional FPN1 knockout (Figure S5). We then measured hepcidin expression in the cortex and hippocampus of *Fpn1*^*Gfap*^ cKO and control mice. The *Fpn1*^*Gfap*^ cKO mice exhibited an increased level of hepcidin compared to that of the *Fpn1*^flox/flox^ mice in both cortex and hippocampus (Fig. 6A). In addition, we detected the expression of hepcidin in astrocytes by double-labelling immunofluorescence, and found that the expression of hepcidin in astrocytes significantly increased (Fig. 6B). Moreover, the FPN1 on BMVECs is reduced (Fig. 6C), indicating lower influx of iron into the brain tissue, whereas iron accumulates inside of the BMVECs as suggested by the increased co-localization of FtL and BMVECs (Fig. 6D).

We next assessed the brain iron content in aged *Fpn1*^*Gfap*^ cKO mice and the control *Fpn1*^flox/flox^ mice. Compared with *Fpn1*^flox/flox^ mice, significantly decreased iron content was detected in the cortex and hippocampus of aged *Fpn1*^*Gfap*^ cKO mice by Perl’s iron staining (Fig. 6E). The concentrations of both FtL and FtH proteins were also reduced in the cortex and hippocampus of *Fpn1*^*Gfap*^ cKO mice (Fig. 6F). The immunofluorescence studies also showed significantly reduced expression of FtL in neurons of *Fpn1*^*Gfap*^ cKO mice (Fig. 6G). These results indicated that ablation of FPN1 in astrocytes led to the decrease of iron contents in the cortex and hippocampus of aged mice. In addition, we found that apoptotic cells were reduced in the cortex and hippocampus of aged *Fpn1*^*Gfap*^ cKO mice (Figure S6A–D), whereas the aggregations of Aβ and phosphorylated tau levels were decreased significantly in aged *Fpn1*^*Gfap*^ cKO mice (Fig. 6H and I, and Figure S6E). These observations may be attributed to the decreased iron level in *Fpn1*^*Gfap*^ cKO mice. To assess if the mitigation of iron accumulation in brain of *Fpn1*^*Gfap*^ cKO mice could alleviate aged associated cognitive decline, we performed behavioral tests by MWM to determine the spatial learning and memory activity. As shown in Fig. 6J–M, the *Fpn1*^*Gfap*^ cKO mice showed significant better learning and memory activity than the control mice. From the above observations, we conclude that downregulation of FPN1 in astrocyte affects its intracellular iron level, triggering hepcidin secretion, which consequently modulates brain iron uptake, and finally influences the function of neurons and improves the cognitive function of mice.

## Discussion

Accumulating evidence has suggested that iron deficiency in the brain can lead to neurological developmental disorders that seriously impact language acquisition, physical activity, and cognition in infants [2, 3]; while iron overload in the brain has been implicated in the pathogenesis of neurodegenerative diseases such as AD and PD [14, 25, 26]. Brain iron content increases with aging, which in turn results in brain iron accumulation especially in the microglia and astrocytes in the cortex and hippocampus [27], the regions that are vulnerable for cognitive function [28]. Due to the double-edged sword nature of iron in the brain, iron metabolism needs to be closely regulated to maintain the normal brain function. The BBB is the key entry site for iron into the brain; hence we considered it crucial to clarify the specific mechanism and regulation mechanism of iron transport through the BBB.

The iron transporter FPN1 is the currently only identified mammalian transporter on the cell membrane that exports iron out of cells [4, 12]. Therefore, the FPN1 on the endothelial cell membrane was suspected to play an essential role in iron crossing the BBB. Using mice with conditionally-ablated FPN1 in capillary endothelial cells, we now confirmed this hypothesis in vivo. As we observed, iron could not be efficiently transported into the brain tissue without FPN1 on abluminal membrane of BMVECs, resulting in an iron-deficient status in *Fpn1*^*Cdh5*^ cKO mice. These findings implied that FPN1 in BMVECs indeed plays a critical role in iron transport across the endothelial cells into the brain. To the best of our knowledge, this is the first direct in vivo evidence showing that FPN1 in BMVECs plays an important role in iron transport into the brain.

How is the expression of FPN1 regulated in BMVECs? Outside of the brain, the hepcidin-FPN1 pathway regulated iron transport across the abluminal membrane of enterocytes in the gut [29–31]. Hepcidin is a small peptide mainly expressed in the liver, and its expression is upregulated by iron overload and is downregulated by iron deficiency [32–34]. Hepcidin binds to the FPN1 and causes its internalization and degradation, thereby reducing cellular iron efflux [31, 35–37]. This regulatory pathway controls the intestinal iron absorption and iron recycling by macrophages [32, 33], maintaining the homeostasis of the systemic iron metabolism. It has been proposed that, similar as the regulatory role of hepatic hepcidin, the astrocyte-derived hepcidin may interact with FPN1 of BMVECs in the brain, thereby regulating brain iron uptake [19, 38, 39]. To explore the role of astrocyte hepcidin in the downregulation of FPN1 in BMVECs in vivo, we generated a mouse model in which hepcidin was selectively knocked down in astrocytes. We found that the expression of FPN1 in BMVECs increased significantly in sh*Hamp* mice, and iron levels increased in different brain regions. These findings together suggest that the astrocyte hepcidin indeed affects FPN1 level on the membrane of BMVECs, which may in turn control iron uptake at the BBB.

To reveal whether the astrocyte hepcidin directly regulates FPN1 expression in BMVECs, we confirmed the reported regulatory effect of hepcidin on FPN1 level by injecting exogenous hepcidin into mouse brain, and by using *Hamp* KO mice to detect the alterations of FPN1 level. We then designed several in vitro experiments to observe the interactions between astrocyte hepcidin and FPN1 on the membrane of BMVECs. To our surprise, we found that treating endothelial cells with medium conditioned by primary astrocytes failed to reduce the expression of FPN1 in endothelial cells. As it has been reported, hepcidin level in healthy humans is very low in cerebrospinal fluid (0.21 to 3.54 ng/mL) [40], as compared to that in the blood (4.95–45.63 ng/mL) [41]. These measurements indicate that the low level of hepcidin secreted into the brain tissue is unlikely to regulate endothelial FPN1 expression in the same way as the circulating hepcidin regulates FPN1 in intestinal epithelial cells.

Based on the structure of BBB [42], we hypothesized that astrocyte hepcidin locally secreted into the gap between the astrocyte endfoot and BMVEC may directly regulate FPN1 of BMVECs. Using co-culture techniques and transwell experiments, we found that astrocyte hepcidin decreased the expression of FPN1 in BMVECs only where astrocytes and BMVECs were in interfacial contact. When hepcidin was depleted in the astrocyte, the FPN1 expression in BMVECs was not affected in the regions of cell-to-cell interaction. In contrast, FPN1 expression in BMVECs was significantly decreased when hepcidin was overexpressed in astrocytes, particularly at the points of contact. In addition, immuno-EM also detected astrocyte hepcidin secreted into the gap between the astrocyte endfeet and BMVECs. These results support the direct regulatory effect of astrocyte hepcidin on FPN1 of BMVECs.

Can astrocytes respond to the change of its intracellular iron level and modulate the expression of hepcidin accordingly? To alter the iron concentration in astrocytes, we studied the *Fpn1*^*Gfap*^ cKO mouse model with conditional knockout of FPN1 in astrocytes. We found that the elevated iron levels in these astrocytes induced a high expression of hepcidin likely via activation of STAT-3 signaling pathways as reported previously [6, 43], and meanwhile the FPN1 of endothelial cells decreased. These findings implied that, similar to the role of hepatocytes in the peripheral system, astrocytes indeed play an important role in sensing iron concentrations and regulating iron influx into the brain.

Furthermore, as brain iron level is closely associated with cognitive function, especially in cortex and hippocampus [1, 44]. Promisingly, we found that knockdown of astrocyte FPN1 increased hepcidin level of *Fpn1*^*Gfap*^ cKO mice, which in turn attenuated iron accumulation in the brain and therefore improved aging-related cognitive function decline. In addition, the sh*Hamp* mice showed a significant decline in cognitive function, which were consistent with the increase of iron content in the hippocampus and cortex in sh*Hamp* mice. These indicate that modulation of brain iron homeostasis by regulating astrocyte hepcidin expression or other iron regulatory molecules may be potential therapies for treating neurodegenerative diseases.

**Fig. 7:**
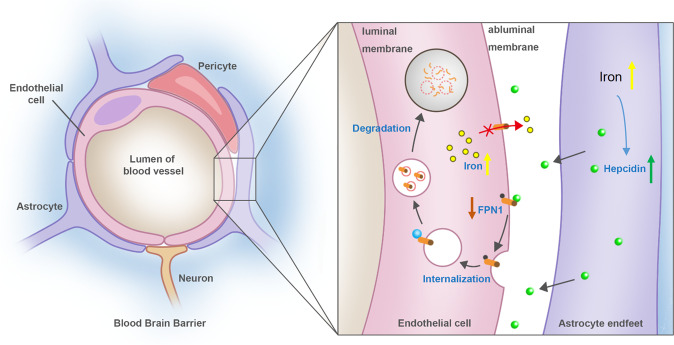
The working model of iron transport across BBB and underlying regulation. Endothelial cells and the surrounding astrocytes are two major components of BBB, where hepcidin and FPN1 are two key molecules in controlling iron transport across BBB into brain parenchymal tissues. In response to the elevated brain iron level, more hepcidin in the astrocyte secreted through its endfeet to directly decrease FPN1 level of BMVECs, which decreased the iron influx from blood into brain tissues and therefore kept the iron homeostasis.

In summary, our results showed FPN1 of BMVECs plays an important role in the process of iron transport across the abluminal membrane of BMVECs into the brain. Hepcidin secreted by astrocytes directly regulates FPN1 in BMVECs at the astrocyte endfeet, thereby controlling the transport of iron into the brain. Furthermore, astrocytes responded to the high intracellular iron levels and raised the secretion of hepcidin subsequently, which in turn modulated total brain iron level through the hepcidin-FPN1 pathway. Thus, FPN1 is a gateway for iron transport into the brain, and the controller of this gateway is astrocyte hepcidin (Fig. 7 and cover image). This study unravelled the intermolecular interaction of iron uptake at the blood-brain barrier, and provided potential therapeutic targets, such as astrocyte hepcidin and microvascular endothelial FPN1, for the treatment of brain iron dysregulation and its related neurological diseases.

## Methods and materials

### Animals

All procedures were carried out in accordance with the Guide for the Care and Use of Laboratory Animals issued by the National Institutes of Health and were approved by the Animal Care and Use Committee of Hebei Science and Technical Bureau in the PRC. All experimental animals were housed in plastic cage at 21 ± 2 °C, and provided free access to food and water. Rooms were humidity controlled and maintained under cycles of 12 h of light and 12 h of dark. Mice were randomly assigned for the following experiments, and littermate controls were used whenever possible. No blinding was carried out for animal experiments.

The GFAP-Cre transgenic mice on a C57BL/6 genetic background were gifted by Dr. Jiulin Du of the Shanghai Institute of Neuroscience. The genotype of GFAP-Cre mice was determined using primer-specific PCR. The primer sequences were as follows: 5’-GCGGTCTGGCAGTAAAAACTATC-3’ and 5’-CCTTCCAGGGCGCGAGTTGATAGCT-3’. The VE-Cadherin-Cre (Cdh5-Cre) transgenic mouse on a C57BL/6 genetic background used in the study were gifted by Dr. Xiao Yang of the Beijing Institute of Lifeomics and were characterized previously [45].

The genotype of Cdh5-Cre mice was determined using primer-specific PCR. The primer sequences were as follows: 5’-GCGGTCTGGCAGTAAAAACTATC-3’ and 5’-GTGAAAC AGCATTGCTGTCACTT-3’. The *Fpn1*^flox/flox^ mice on a 129/SvEvTac genetic background utilized in this study were also characterized previously [46]. The genotype of *Fpn1*^flox/flox^ mice was determined using primer-specific PCR. The primer sequences were as follows: 5’-GGTTAAAC TGCTTCAAAGG-3’ and 5’- CTACACGTGCTCTCTTGAGAT-3’. Male transgenic mice were used in all the experiments. The *Hamp* KO mice were described previously [47]. The coding exons 1 and 2 have been knocked out. The genotype of *Hamp* KO mice was determined using primer-specific PCR. The primers were as follows: 5’-CCAAACAAAAGTGTTCCTTGG-3’; 5’-GACCTGTAAA CCCAGCTCAG-3’; and 5’-GCAGCGCATCGCCTTCTATC-3’.

Mice with preferential knockdown of hepcidin in astrocytes were generated by Cyagen Biosciences Inc (Guang Zhou, China) as described previously [6]. The vector pRP.ExSi-GFAP-sh*Hamp*-1 (GFAP promoter—human pre-miR30a flanking sequence—sense—loop—antisence (GCAGACATTGCGATACCAATAGTGAAGCCACAGATGTATTGGTATCGCAATGTCTGA)—human pre-miR30a 3’ flanking sequence-bGH polyA) was microinjected into a fertilized egg. Recombinant embryonic stem cells were injected into FVB blastocysts to produce chimaeras, which were then crossed with FVB mice to produce homozygous mice of preferential knockdown of hepcidin in astrocytes (GFAP-sh*Hamp*). The genotype of GFAP-sh*Hamp* mice was determined using primer-specific PCR. The primer sequences were as follows: PCR primer forward 5’-AGCTTTATTGCGGTAGTTTATCACA-3’ and reverse 5’-AAAGTAGCCCCTTGAATTCCGA-3’. Male transgenic mice were used in all the experiments.

### Generation of astrocyte *Hamp* and *Fpn1* overexpression mice

The full-length mouse *Hamp* coding sequence was amplified by PCR (primers: 5’-ATGGCACTCAGCACTCGG-3’ and 5’-CTATGTTTTGCAACAGATACCA-3’) and cloned into the pIRES2-DsRed-Express vector. The human GFAP promoter was amplified by PCR (primers: 5’-GAGCTCCCACCTCCCTCTC-3’ and 5’-CCTGCTCTGGCTCTGCTC-3’) and used to replace the CMV promoter to generate pIRES2-GFAP-*Hamp*. The full-length mouse *Fpn1* coding sequence was amplified by PCR (primers: 5’-CCGGAATTCATGACCAAGGCAAGAGATCAAACCCA-3’ and 5’-GAAGATCTCTATTTTTATACAACAGATGTATTCGG-3’) and inserted into the MCS of pAAV plasmid. The CMV promoter was replaced by hGFAP promoter (primers: 5’-CCGACGCGTGAGCTCCCACCTCCCTCTCTG-3’ and 5’-CCGGAATTCTCACCTGCTCTG GCTCTGCTCGCT-3’). This expression plasmid was named as pAAV-GFAP-*Fpn1*. The pAAV-GFAP backbone vector without inserting *Fpn1* was used as control. The animals were deeply anesthetized with nembutal by intraperitoneal injection, and then 2.5 μg of pIRES2-GFAP-*Hamp* or pAAV-GFAP-*Fpn1* plasmid was injected into the right lateral cerebral ventricle. After intracerebroventricular (ICV) injection, a conductive gel (Siemens) was applied to the skin of the skull, followed by electroporation with genepaddles (5 × 7 mm gold plated) using an ECM 830 square-wave electroporation system (BTX, Division of Genetronics, Inc., San Diego, CA, USA). The voltage, interval cycle length, interval pause and times were 150 V, 50 ms, 100 ms and 5, respectively.

### Brain capillary isolation

Brain capillaries were isolated as previously described [7], with slight modifications. Briefly, the brain was weighed and homogenized in a buffer solution with Ficoll 400 (40%) at ice-cooling water condition (sample: buffer: Ficoll 400 = 1: 3: 4 volume ratio). The buffer solution contained: HEPES, 10 mM; NaCl, 141 mM; KCl, 4 mM; MgSO_4_, 1.0 mM; NaH_2_PO_4_, 1.0 mM; CaCl_2_, 2.5 mM; glucose, 10 mM; and sodium pyruvate, 1 mM, at pH 7.4. The homogenate was then centrifuged at 5800 *g* for 20 min in an Eppendorf centrifuge at 4 °C. The pellet was carefully separated from the supernatant. The pellet was then washed with the buffer and prepared for western blot. Light microscopic examination confirmed that the pellet consisted mainly of networks of brain vessels.

### Primary cell culture

Primary cultured mouse glial cells were prepared by the method of Shao et al. with slight modification [48]. Briefly, the cortical tissue of newborn hepcidin-deficient or normal C57BL/6 mice was dissected in ice-cold D-Hanks’ solution. Blood vessels and piamater were thoroughly removed from the cortical, and the remaining cortex and hippocampal tissue was digested in 10 mL 0.125% trypsin solution at 37 °C for 15 min. After digestion, DMEM/F12 (GIBCO, Thermo Fisher Scientific, MA, USA) culture medium containing 20% heat-inactivated fetal bovine serum (FBS) was added to the mixture, and the sample was filtered through a 100 μm cell strainer (BD Falcon, USA). The cell suspension was then centrifuged and re-suspended in DMEM/F12 containing 10% FBS. The cells were plated at a density of 5×10^7^ cells per poly-L-Lysine-coated 75 cm^2^ flask (Corning, Corning Incorporated, NY, USA) and grown in DMEM/F12 with 10% FBS, 100 U/mL of penicillin, and 0.1 mg/mL of streptomycin at 37 °C in a humidified atmosphere containing 5% CO_2_. The medium was changed every 2 days, and the cells were allowed to reach 90% confluence. For primary cultured astrocytes, mixed glial cultures at day 10 were shaken by rotatory shaking (220 rpm, 24 h) to remove non-astrocyte cells.

### Co-culture of astrocytes and bEND.3 cells

To distinguish them from the primary astrocytes, the bEND.3 cells (ATCC, No. CRL-2299) were incubated with 5-(and 6)-carboxyfluorescein diacetate succinimidyl ester (CFSE, 10 μM). The stained bEND.3 cells were cultured alone or with astrocytes at a 1:1 ratio in 12-well plates. After incubating for 24 h, the CFSE-positive bEND.3 cells were sorted and collected using fluorescence-activated cell sorting (FACS). The FPN1 expression was then detected in sorted bEND.3 cells by western blot analysis.

### Astrocyte and bEND.3 cell co-culture using transwell inserts

The transwell approach is a widely utilized method for cell co-culture [11]. Three different models were prepared. For model 1, only bEND.3 endothelial cells (40,000 cells/cm^2^) were cultured on the inside of the collagen-coated polycarbonate membrane (0.4 μm) of the transwell (12-well type, Corning, Corning Incorporated, NY, USA). For model 2, purified primary astrocytes (20,000 cells/cm^2^) were seeded in the petri dish. After one day of culture, bEND.3 endothelial cells (40,000 cells/cm^2^) were seeded on the inside of the transwell insert, which was placed in the petri dish. For model 3, purified primary astrocytes (20,000 cells/cm^2^) were cultured on the outside of the polycarbonate membrane of the transwell, and directed upside down in the well. After one day of culture, bEND.3 endothelial cells (40,000 cells/cm^2^) were seeded on the inside of the transwell insert to create a sandwich configuration with bEND.3 in the upper layer, the transwell membrane in the middle layer, and astrocytes in the lower layer. The culture medium (0.5 mL on the insert and 1.5 mL in the outer well) was replaced every other day. The endothelial cells were harvested for detection of FPN1 expression by western blot analysis.

### RNA isolation and quantitative PCR

Primary cultured cells were collected in TRIzol reagent (Invitrogen, Thermo Fisher Scientific, MA, USA). The cDNA was synthesized from 1 μg of the extracted total RNA by using a M-MLV Reverse Transcriptase kit (Takara) according to the manufacturer’s protocol. The level of *Hamp*1 mRNA was assessed by quantitative PCR as previously described [12]. PCR amplification was performed with SYBR® Green PCR Master Mix (Applied Biosystems, USA) via a BioRad real-time PCR system with the following cycling parameters: 95 °C for 10 min, followed by 40 cycles of 95 °C for 5 s and then 60 °C for 10 s. β-actin was used as a housekeeping gene. Primer sequences for β-actin were as follows: β-actin forward 5’-AGGCCCAGAGCAAGAGAGGTA-3’ and reverse 5’-TCTCCATGTCGTCCCAGTTG-3’. The primer sequences for hepcidin were as follows: hepcidin forward 5’-TTGCGATACCAATGCAGAAGAG-3’ and reverse 5’-AATTGTTACAGCATTTACAGCAGAAGA-3’. The cycle time (Ct) values for the gene of interest were first normalized with β-actin in the same sample, and then the relative differences between the control and each of the other groups were calculated by using the equation 2^-ΔΔCt^, and expressed as relative fold changes of the control group.

### Injection of isotope iron

Each mouse was treated by ICV injection of 0.8 μg/μL ^57^FeCl_3_ for 5 μL or by intravenous injection of 163.5 μg/μL ^57^FeCl_3_ for 100 μL. 24 h after injection, the mice were perfused intracardially with saline and the brains were isolated and digested for ICP-MS.

### Inductively coupled plasma mass spectrometry (ICP-MS)

The total iron contents of the cortex, hippocampus and isolated BMVECs were determined by ICP-MS as previously described [8, 9]. Approximately 10 mg of the tissue was added to 2 mL 70% ultrapure nitric acid (15 M, J.T. Baker, USA) in Teflon digestion tubes, and digested in a microwave digestion system for 2 h at 100 °C, and then 4 h at 200 °C [8]. To set up a standard curve for Fe, a stock solution of 2 ng Fe in 10 µL in 2% HNO_3_ was diluted to 0.25 ng/10 µL, 0.5 ng/10 µL, 0.75 ng/10 µL, and 1 ng/10 µL in 1% HNO_3_. The standards and digested samples were assessed in triplicate by ICP-MS.

### Perl’s staining

3, 3’-Diaminobenzidine tetrahydrochloride (DAB)-enhanced Perl’s staining was performed as previously described [10]. Briefly, 15 µm sections were washed, and treated with 0.1 M Tris-buffer saline (TBS, pH 7.4) containing 3% hydrogen peroxide (H_2_O_2_) for 10 min. The brain sections were then immersed in Perl’s solution containing equal amounts of fresh preparation, aqueous potassium ferrocyanide (2%) and hydrochloric acid (2%) for 6 h. After washing, the sections were finally intensified with DAB. Finally, the slides were rinsed, dehydrated, covered with neutral balsam and examined under a light microscope.

### Synchrotron radiation X-ray fluorescence (SR-XRF)

The distributions of iron in mouse brain samples were detected in fluorescence mode at room temperature at the 4W1B end-station of Beijing Synchrotron Radiation Facility (BSRF). Brain sections were cut at 30 μm intervals on a freezing microtome. Equivalent slices from the same coordinates were fixed onto 3 mm-thick Mylar films (polycarbonate) from each of the groups of mice, dried at room temperature and stored in a vacuum desiccator before analysis by SR-XRF. The continuous synchrotron X-rays were monochromatized by a Si (111) double crystal. The sample was placed at a 45° angle to the incident X-ray beam, and X-ray fluorescence was detected with a 50 mm^2^ silicon drift detector (Vortex, Hitachi High-Technologies Science America Inc, Northridge, CA, USA) oriented at a 90° angle to the incident beam. A light microscope was coupled to a computer for sample viewing. A monochromatic SR with photon energy of 10 keV was used to excite the samples. The beam was adjusted with a grating to about 50 × 50 μm^2^. The sample platform was moved at intervals of 150 μm. The XRF signals were collected for up to 12 s at each point. In order to correct the effect of the SR beam flux variation on the signal intensity, the peak areas of Fe were normalized to the current intensity (I0) in an ionization chamber, which was placed in front of the samples. The areas under the peaks were used for estimating the relative iron content in the samples. The results were analyzed using Origin 8.0 (Northampton, MA, USA), and the iron signals in different brain regions were compared among the different groups.

### Behavioral test

The Morris Water Maze test (MWM test) was used to assess spatial learning and memory deficits as described previously [10]. At first, a visible platform test was performed, which confirmed that there were no significant differences in sensory, motor, or motivational activities among different groups of mice. Next, hidden platform tests were performed in succession. The test was conducted four times a day for four days, with four randomized starting points. The position of the escape platform was kept constant. Each trial lasted for 120 s or ended as soon as the mouse reached the submerged platform. The platform was removed on the fifth day, and the number of times that the mouse crossed the platform in 120 s and the time spent in the target quadrant were recorded for analysis.

### Western blot analysis

Protein expression was assessed by western blot analysis following polyacrylamide gel electrophoresis as previously described [48, 49]. The blots were incubated with a rabbit anti-mouse FtH/L (1:1000; ab75973, Abcam, USA) or rabbit anti-FPN1 (1:5000; MTP11-A, Alpha Diagnostics International, USA), or β-actin (A5060, Sigma-Aldrich St. Louis, USA) primary antibody overnight at 4 °C. The membrane was washed four times for 15 min each with Tris-buffered saline solution containing 0.05% Tween-20 and incubated with an goat anti-rabbit (RPN4301, Amersham, UK) or goat anti-mouse secondary antibody (RPN4201, Amersham, UK) conjugated to horseradish peroxide (1:5000) for 90 min at room temperature. Peroxidase activity was detected with the SuperSignal™ West Pico PLUS Chemiluminescent Substrate (Thermo Fisher Scientific Inc, Waltham, USA) and visualized with ImageQuant (Fujifilm LAS-4000, Tokyo, Japan). The optical densities of the bands were analyzed using Multi Gauge software (V3.1; Fujifilm, Tokyo, Japan). The relative band intensities of the proteins were calculated in comparison to that of β-actin. All experiments were performed at least three times.

### Immunohistochemistry

Immunohistochemistry was performed as previously described [49]. Serial coronal sections were cut at a thickness of 15 µm on a freezing microtome and mounted onto a slide covered with 3-aminopropyl-triethoxysilane (APES, Beijing ZhongShan Biotechnology, Beijing, China). After blocking for 1 h with normal goat serum prepared in 0.01 M PBS, the slices were incubated overnight at 4 °C with a mouse anti-GFAP monoclonal antibody (1:400) (MAB360, Millipore Corporation, USA), anti-NeuN monoclonal antibody (1:100) (MAB377, Millipore Corporation, USA), rabbit anti-FPN1 antibody (1:400) (MTP11-A, Alpha Diagnostic International, USA), rabbit anti-hepcidin polyclonal antibody (1:300) (ab75883, Abcam, USA), rabbit anti-FtH (1:400) (ab65080; Abcam, USA), goat anti-PECAM-1/CD31 (1:200) (BM1346, Boster, USA), Rabbit anti-beta Amyloid (1:1000) (ab201060; Abcam, USA) or rabbit anti-phospho Tau (1:400) (12885; Cell Signaling Technology,). Negative controls were processed using the same procedures, but normal serum was used in place of the antibodies. For immunofluorescence, the following secondary antibodies were incubated at 37 °C for 60 min: rhodamine-conjugated goat anti-rabbit IgG (1:200) (AP132R, Millipore Corporation, USA), FITC-conjugated goat anti-mouse IgG (1:200) (AP124F, Millipore Corporation, USA) and 647 donkey anti-goat IgG (1:200) (A-21447, Thermo Fisher Scientific, USA). Finally, the sections were photographed with a Zeiss LSM710 microscope. For immunohistochemistry, biotinylated goat anti-rabbit secondary antibody (1:200) (81-6540, Zymed Laboratories, USA) was added and incubated for 60 min at 37 °C. After that, the sections were incubated with streptavidin–horseradish peroxidase conjugate (1:200 dilution; 81-6540, Zymed Laboratories) for 60 min at 37 °C. The slides were rinsed in PBS four times (5 min each time) after every step. Finally, the staining reaction was developed in DAB solution (DAB substrate kit, ZLI-9018, ZSGB-BIO, China).

### Assessment of apoptosis

The degree of apoptosis in the different brain regions of *Fpn*^flox/flox^ and *Fpn1*^*Gfap*^ cKO mice was assessed by the terminal deoxynucleotidyl transferase-mediated FITC-dUTP nick-end labeling method (TUNEL), following the manufacturer’s protocol. Nuclei were counterstained with DAPI. The number of TUNEL-DAPI-positive cells was counted as described previously [6]. The counting area was located in the same position in all groups. For each group, quantification was performed in sections from three different mice.

### Immunoelectron microscopy (immuno-EM)

The detection of the location of hepcidin secreted by astrocytes in 3 month-old mice was performed by immune-EM as described previously [50]. Briefly, small pieces of cortex were fixed in 4% paraformaldehyde with 0.5% / 0.1% glutaraldehyde for 3 h at 4 °C, and the brain samples were dehydrated with increased gradient ethanol at 4 °C. The cortex samples were infiltrated in mixture of ethanol and LR WHITE (London Resin Co., Basingstoke, UK), which were finally polymerized in LR White and kept in room temperature for solidification. Ultrathin sections were cut with the thickness of 100 nm and incubated with rabbit anti-hepcidin polyclonal antibody, and 10 nm immuno-gold conjugated secondary antibody was used in the system. After that, sections were continued to be fixed in 1% glutaraldehyde and stained in 2% uranyl acetate. Finally, all the cortex sections were observed in Hitachi TEM H-7650.

### Thioflavin staining

Thioflavin staining of brain tissue can observe amyloid plaques. At first, brain sections were washed three times for 5 min each in distilled water, and then incubated in darkness in filtered 1% aqueous Thioflavin-S for 8 min at room temperature. After rinsing thoroughly in PBS and dehydrating through a series of graded alcohols (5 min each in 70% and 80% and then 10 min each in 90%, 95%, and 100% ethanol), these sections were immersed in xylene (twice for 10 min each). The treated sections were finally cover slipped with Canada balata. All steps were performed in darkness after thioflavin staining.

### Statistical analysis

All the data are expressed as the mean ± SEM. The statistical analyses of group differences were assessed by a one-way ANOVA followed by post-hoc test (Dunnett test and Student-Newman-Keul’s test), or by students t-tests. Differences were considered significant when *p* < 0.05. All the tests were performed with SPSS 21.0 (SPSS Inc., Chicago, IL).

## Supplementary information

Supplementary Figure S1–S6 and Figure legends

Original Data File

Original Data File

Original Data File

Original Data File

Original Data File

Original Data File

Original Data File

Original Data File

Reproducibility checklist- CDDIS-22-0542R

## Data Availability

All data needed to evaluate the conclusions in the paper are present in the paper. Additional data related to this paper may be requested from the corresponding authors.
