# Supplementary material for: Astrocyte-derived hepcidin controls iron traffic at the blood-brain-barrier via regulating ferroportin 1 of microvascular endothelial cells
Source: Cell Death Dis. 2022 Aug 1;13(8):667. doi: 10.1038/s41419-022-05043-w (PMC9343463; doi:10.1038/s41419-022-05043-w)
Supplement: Supplementary file 1 — Supplementary Figure S1–S6 and Figure legends [file 41419_2022_5043_MOESM1_ESM.docx]

**Supplementary figures**

**Figure S1**

**
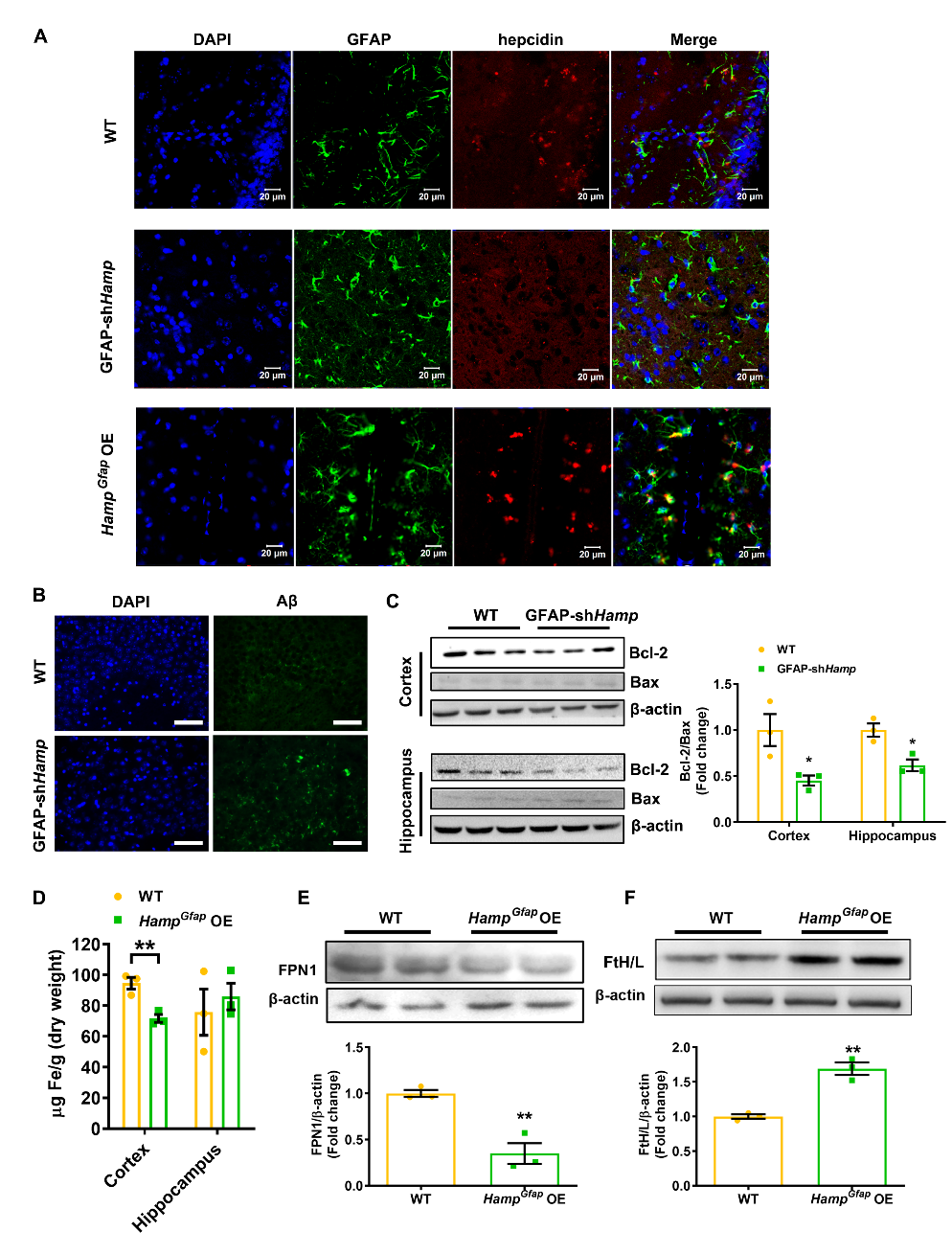
**

**Figure S1. Brain iron levels decreased in *Hamp^Gfap^* OE mice and iron accumulated in BMVECs.** (A) The differences of hepcidin expression in astrocytes of 6-month-old WT, GFAP-sh*Hamp*, and *Hamp*^Gfap^ OE mice were determined by immunofluorescence staining with anti-GFAP (astrocyte marker) and anti-hepcidin (GFAP-sh*Hamp* mice) or anti-DsRed antibody (*Hamp^Gfap^* OE mice). Scale bar = 20 μm. (B) Detection of Aβ (green) by immunofluorescence staining. Scale bar = 50 μm. (C) Detection and analysis of Bcl-2/Bax expression ratio by western blot. Data are presented as the mean ± SEM, n = 3, **p* < 0.05 GFAP-sh*Hamp* vs. WT group. (D) Total iron in the cerebral cortex and hippocampus of 6-month-old *Hamp^Gfap^* OE and control mice were determined by ICP-MS. Data are presented as the mean ± SEM, n = 3, ***p* < 0.01 *Hamp^Gfap^* OE vs. WT group. (E-F) Levels of FPN1 (E) and FtH/L (F) in BMVECs of different mice were detected by western blot. Relative expression levels were normalized by β-actin and expressed as the mean ± SEM, n = 3, ***p* < 0.01 vs. WT group.

**Figure S2**

**
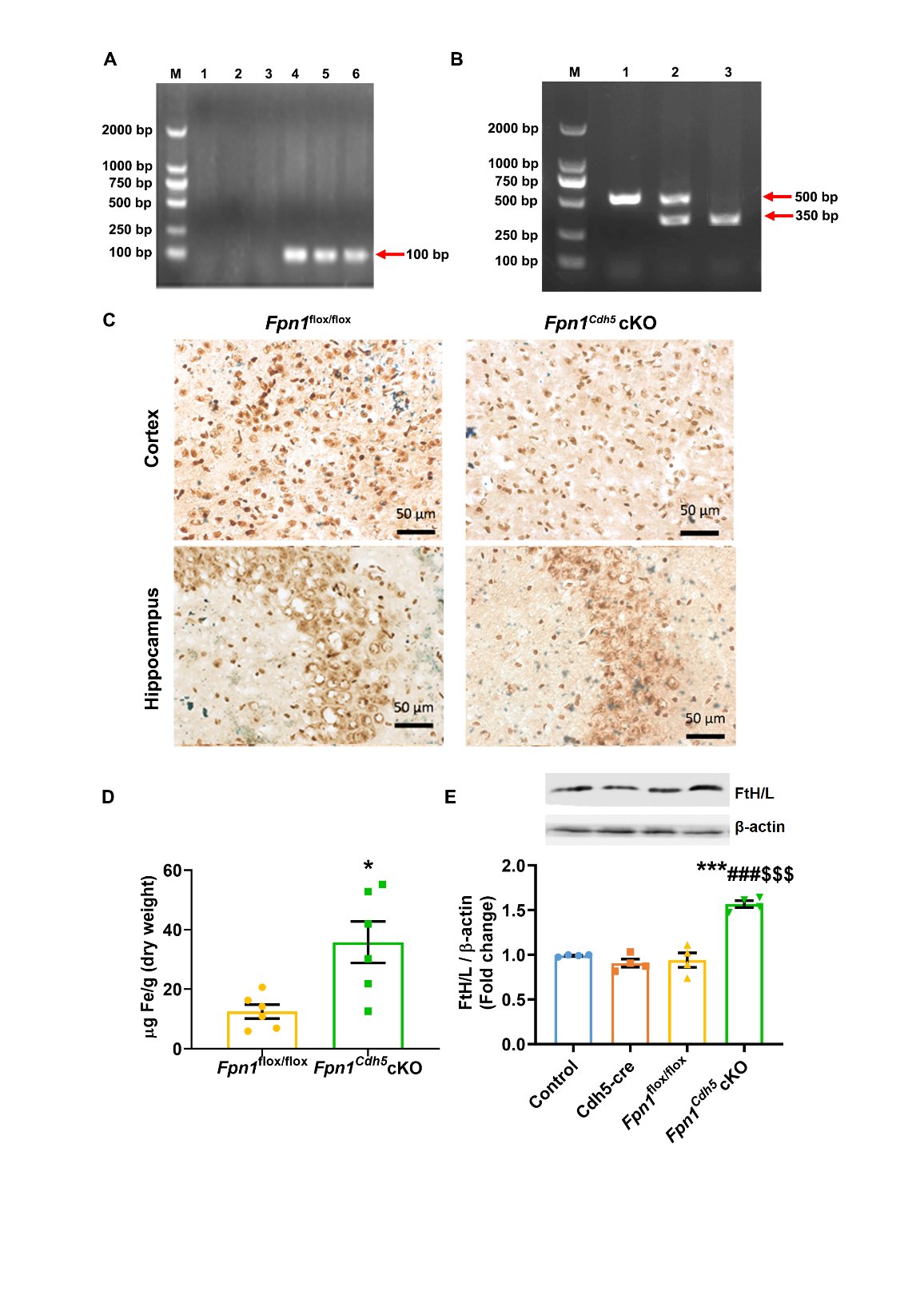
**

**Figure S2. Iron content in cortex and hippocampus decreased and BMVECs’ iron increased in *Fpn1^Cdh5^* cKO mice.** (A-B) The genotype of mice used in this study was identified by primer-specific PCRs. (A) Mice, such as No. 4 to 6, possessing the 100 bp-length PCR products were Cre transgenic mice, while mice, such as No. 1 to 3, without the 100 bp-length PCR products were wild-type mice. M: DNA ladder. (B) Mice, such as No. 1, which possess only the 500 bp-length PCR product, are homozygous for the loxp sites, while those like No. 2, which possess both the 500 bp-length and a 350 bp-length PCR products, are heterozygous transgenic mice. Mice, such as No. 3, that possess only the 350 bp-length PCR products, do not harbor loxp sites. M: DNA ladder. (C) Cortex and hippocampus sections from 9-week-old *Fpn1*^flox/flox^ and *Fpn1^Cdh5^* cKO mice were detected by DAB-enhanced Perl's staining. Representative images are shown. Iron deposits appear as brown granules. Scale bar = 50 μm. (D) Total iron in BMVECs was determined by ICP-MS in *Fpn1^Cdh5^* cKO and control *Fpn1*^flox/flox^ mice. Values are presented as the mean ± SEM, n = 6, **p* < 0.05 vs. *Fpn1*^flox/flox^ group. (E) The level of ferritin (FtH/L) in BMVECs was estimated by western blot analysis. The relative expression levels were normalized to β-actin levels and expressed as the mean ± SEM, n = 4, ****p* < 0.001 vs. control group; ###*p* < 0.001 vs. Cdh5-cre group; $$$*p* < 0.001 vs. *Fpn1*^flox/flox^ group.

**Figure S3**


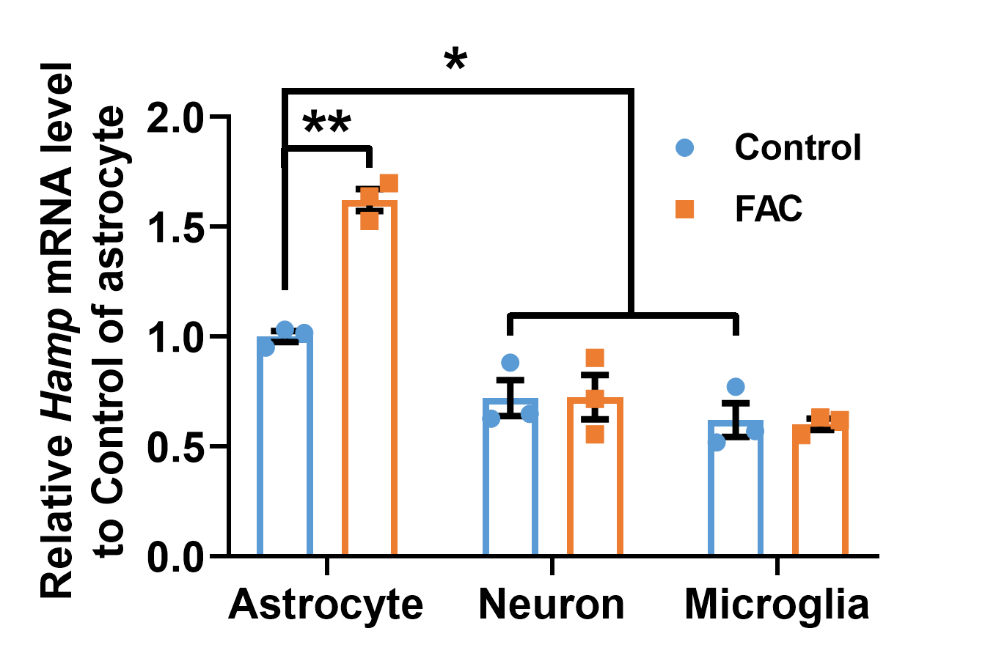


**Figure S3. The mRNA level of *Hamp* was detected by real-time PCR in primary astrocyte, microglia and neuron following FAC treatment**. Relative expression levels were normalized by β-actin and expressed as the mean ± SEM, n = 3, **p* < 0.05 and ***p* < 0.01.

**Figure S4**


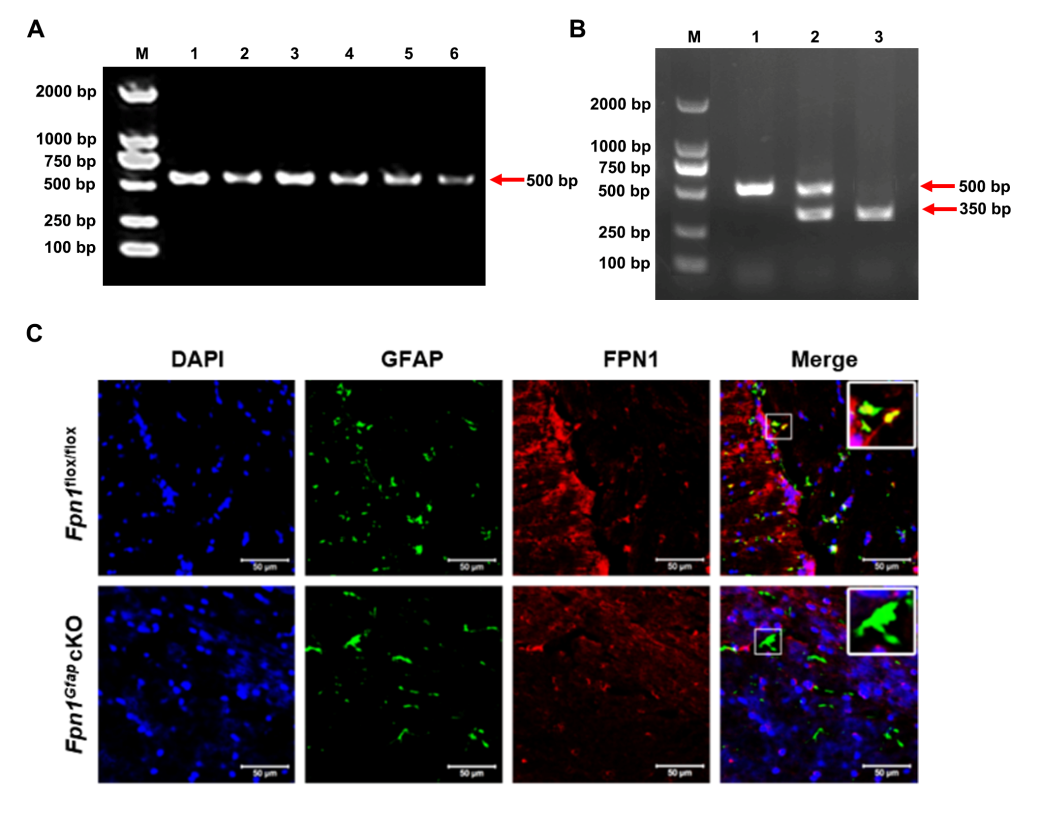


**Figure S4. Identification of transgenic mice *Fpn1^Gfap^* cKO.** (A) Mice, such as No. 1 to 6, possessing the 500 bp-length PCR products were GFAP-Cre transgenic mice, while mice without the 500 bp-length PCR product were wild-type mice. M: DNA ladder. (B) Mice, such as No. 1, which possess only the 500 bp-length PCR products, are homozygous for the loxp sites, while those like No. 2, which possess both the 500 bp-length and a 350 bp-length PCR products, are heterozygous transgenic mice. Mice, such as No. 3, that possess only the 350 bp-length PCR products, do not harbor loxp sites. M: DNA ladder. (C) Double immunofluorescence labelling of FPN1 (red) and GFAP (green) was carried out in *Fpn1*^flox/flox^ and *Fpn1^Gfap^* cKO mice. Scale bar = 50 μm.

**Figure S5**

**
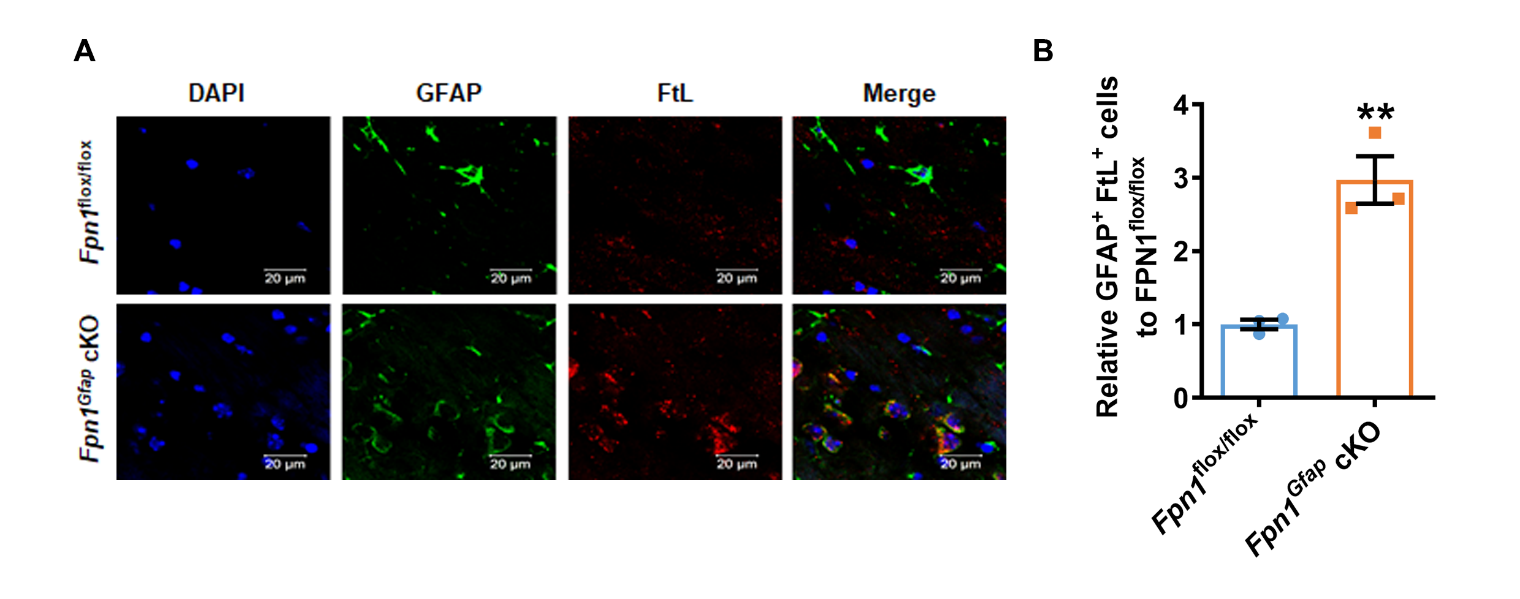
**

**Figure S5. Iron increased in astrocytes but decreased in neurons in the cortex and hippocampus of *Fpn1^Gfap^* cKO mice.** (A) Double immunofluorescence labelling of GFAP (green) and FtL (red) was carried out in 15-month-old *Fpn1*^flox/flox^ and *Fpn1^Gfap^* cKO mice. Scale bar = 20 μm. (B) The relative GFAP and FtL positive cells were counted. Data were expressed as the mean ± SEM, n = 3, ***p* < 0.01.

**Figure S6**


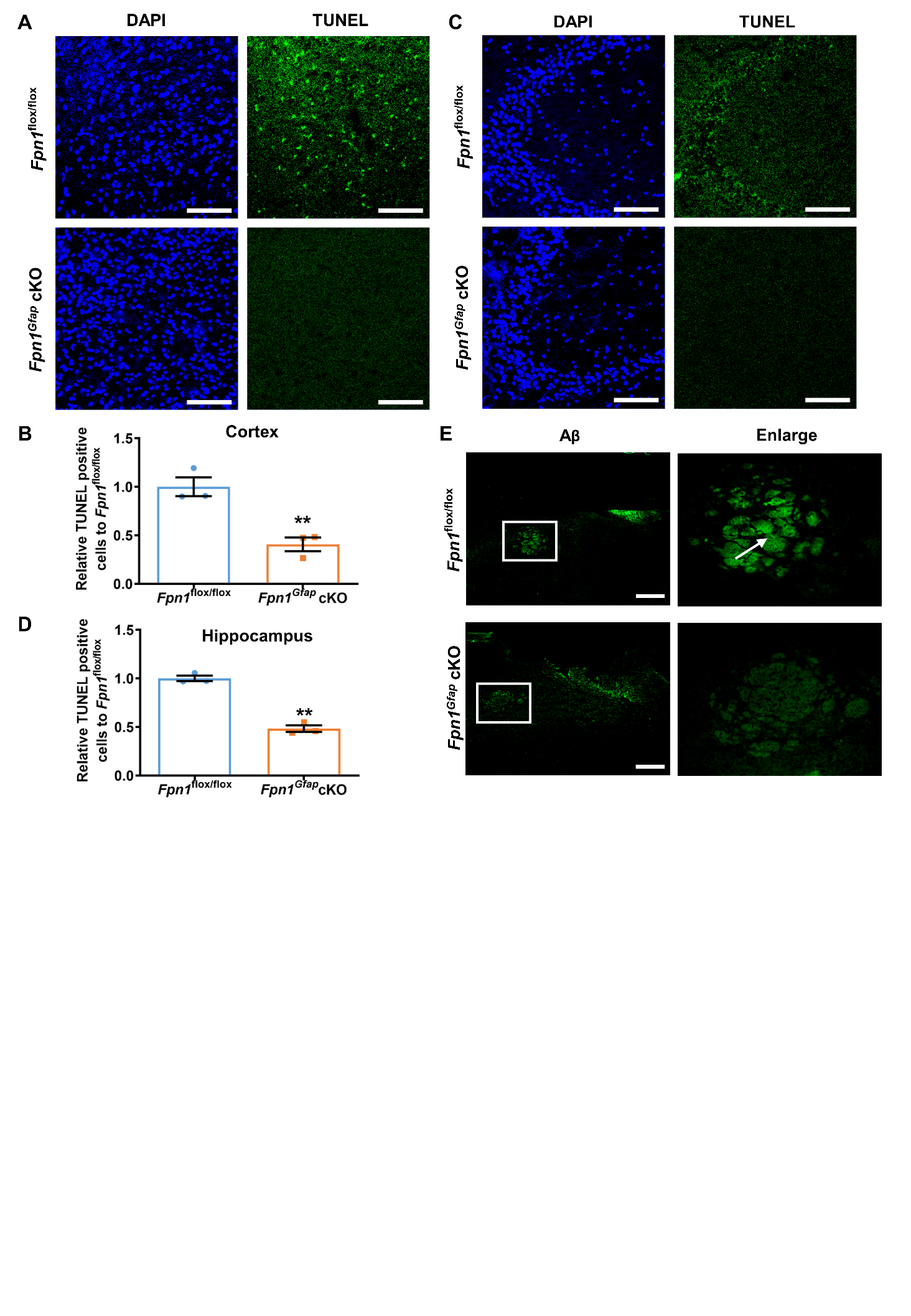


**Figure S6. Knockout of FPN1 in astrocyte relieves the apoptosis of neuron and decreased Aβ aggregation and tau phosphorylation.** (A-D) Apoptosis in cortex (A-B) and hippocampus (C-D) of 15-month-old *Fpn1*^flox/flox^ and *Fpn1^Gfap^* cKO mice were assayed by DAPI and TUNEL staining. Scale bar = 100 μm. The TUNEL- positive cells were counted in five separate fields and are presented as fold changes as compared to the control group. Values are presented as the mean ± SEM, n=3. ***p* < 0.01 vs. *Fpn1*^flox/flox^ mice. (E) The Aβ levels of 15-month-old *Fpn1*^flox/flox^ and *Fpn1^Gfap^* cKO mice were detected by thioflavin staining. Scale bar = 50 μm.
