# Supplementary figures and images for: Astrocyte-derived hepcidin controls iron traffic at the blood-brain-barrier via regulating ferroportin 1 of microvascular endothelial cells
Source: Cell Death Dis. 2022 Aug 1;13(8):667. doi: 10.1038/s41419-022-05043-w (PMC9343463; doi:10.1038/s41419-022-05043-w)

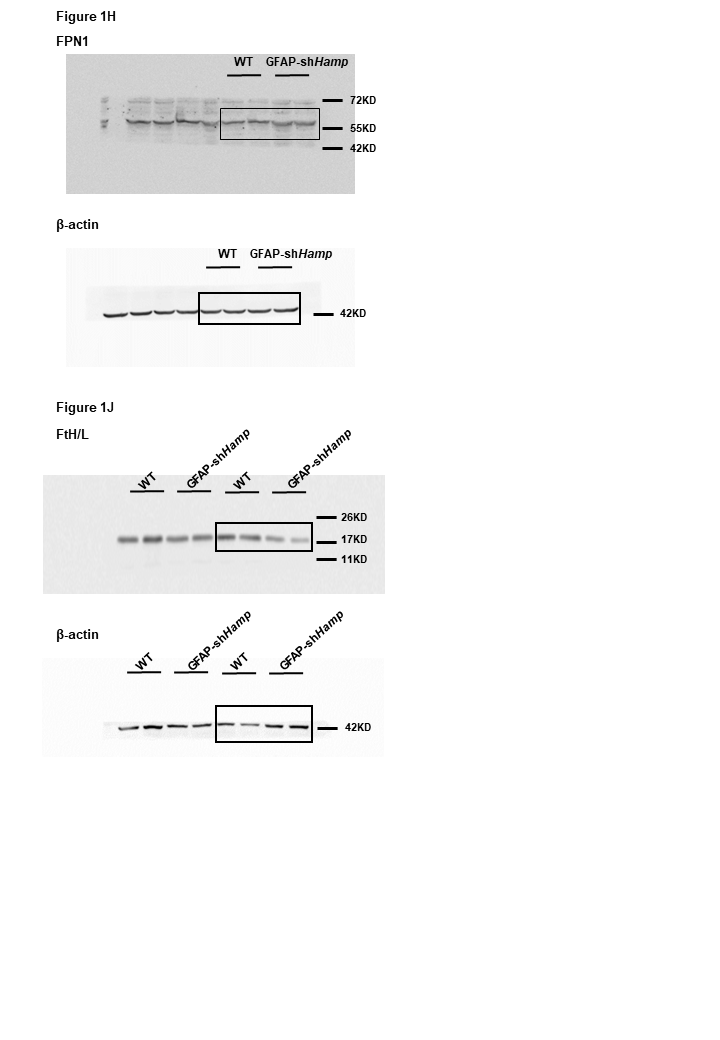

Supplement: Supplementary file 2 — Original Data File [file 41419_2022_5043_MOESM2_ESM.tif]

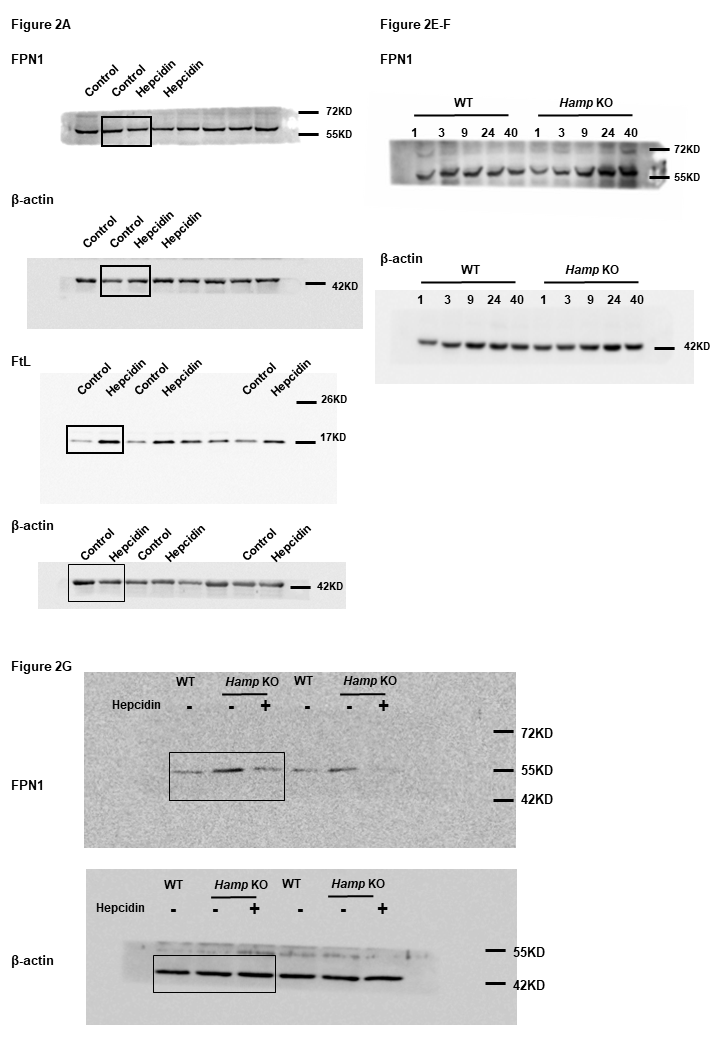

Supplement: Supplementary file 3 — Original Data File [file 41419_2022_5043_MOESM3_ESM.tif]

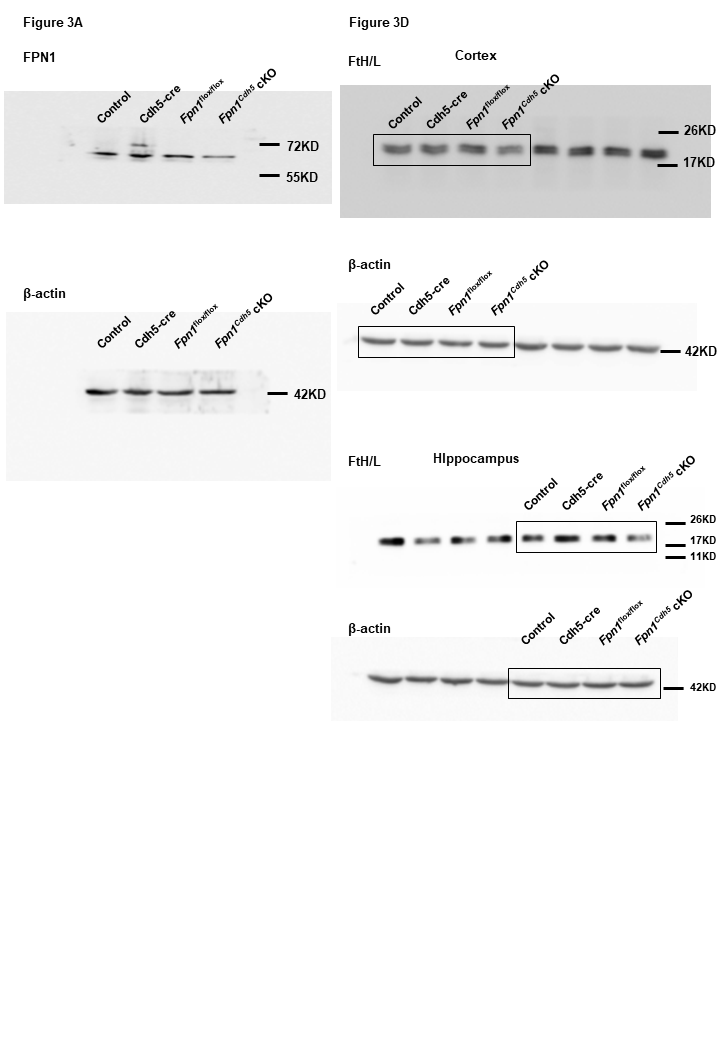

Supplement: Supplementary file 4 — Original Data File [file 41419_2022_5043_MOESM4_ESM.tif]

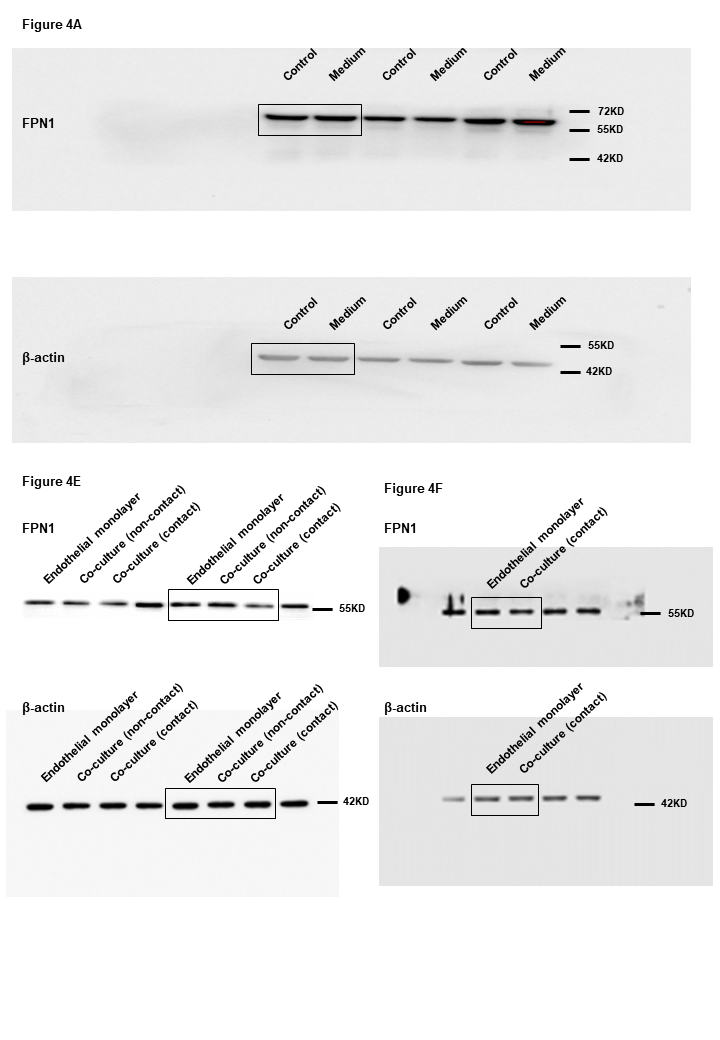

Supplement: Supplementary file 5 — Original Data File [file 41419_2022_5043_MOESM5_ESM.tif]

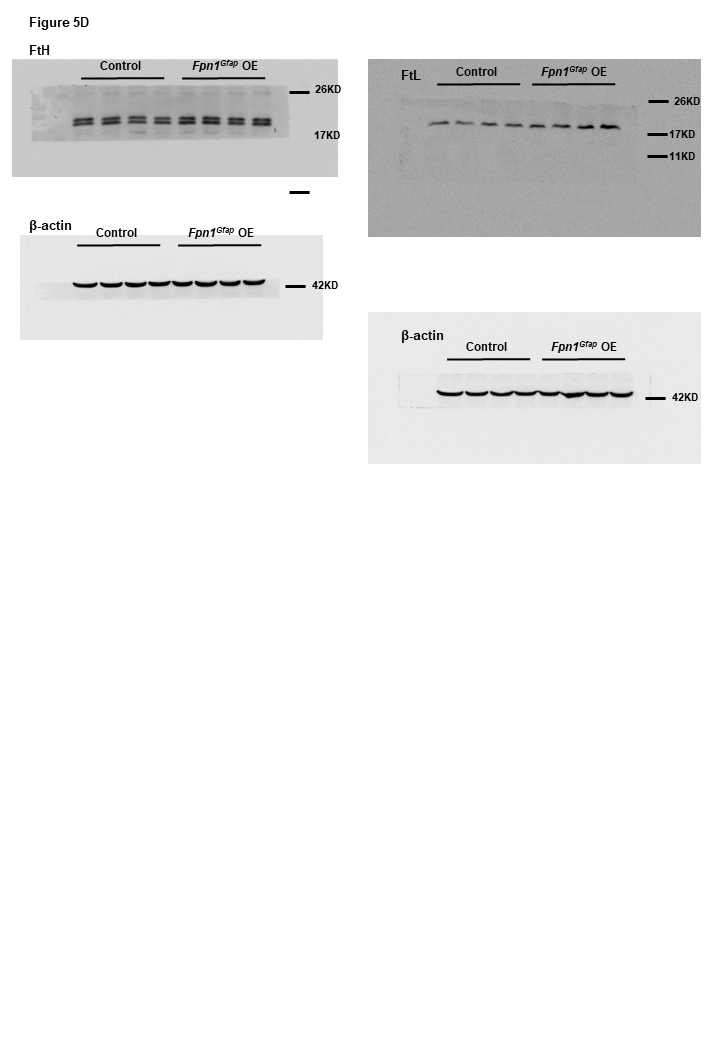

Supplement: Supplementary file 6 — Original Data File [file 41419_2022_5043_MOESM6_ESM.tif]

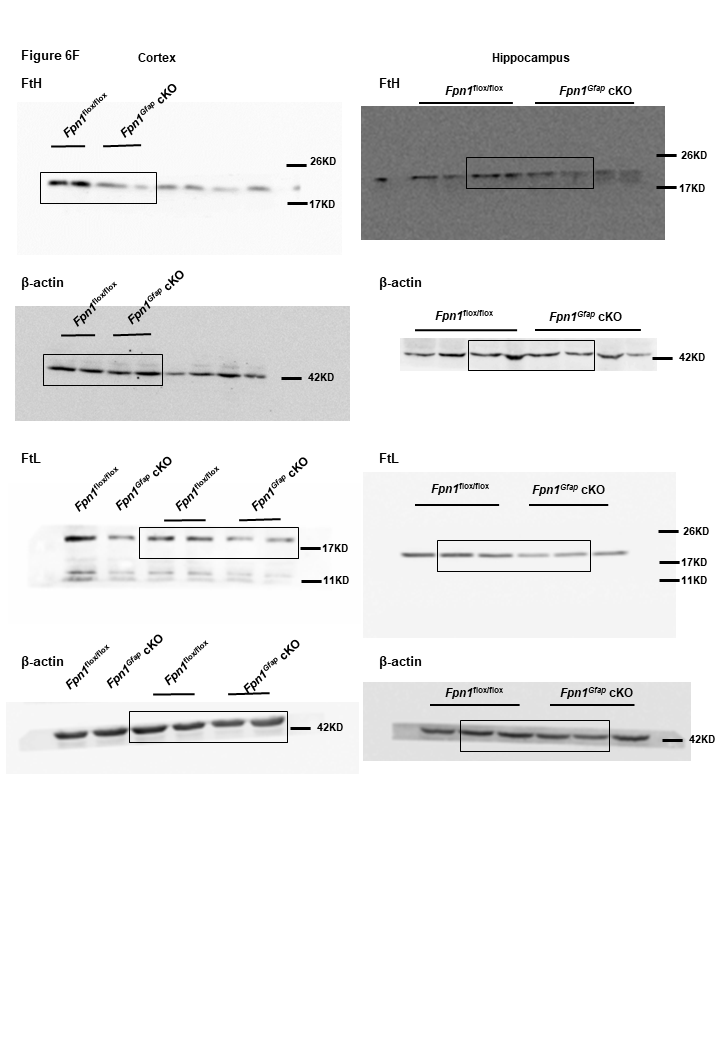

Supplement: Supplementary file 7 — Original Data File [file 41419_2022_5043_MOESM7_ESM.tif]

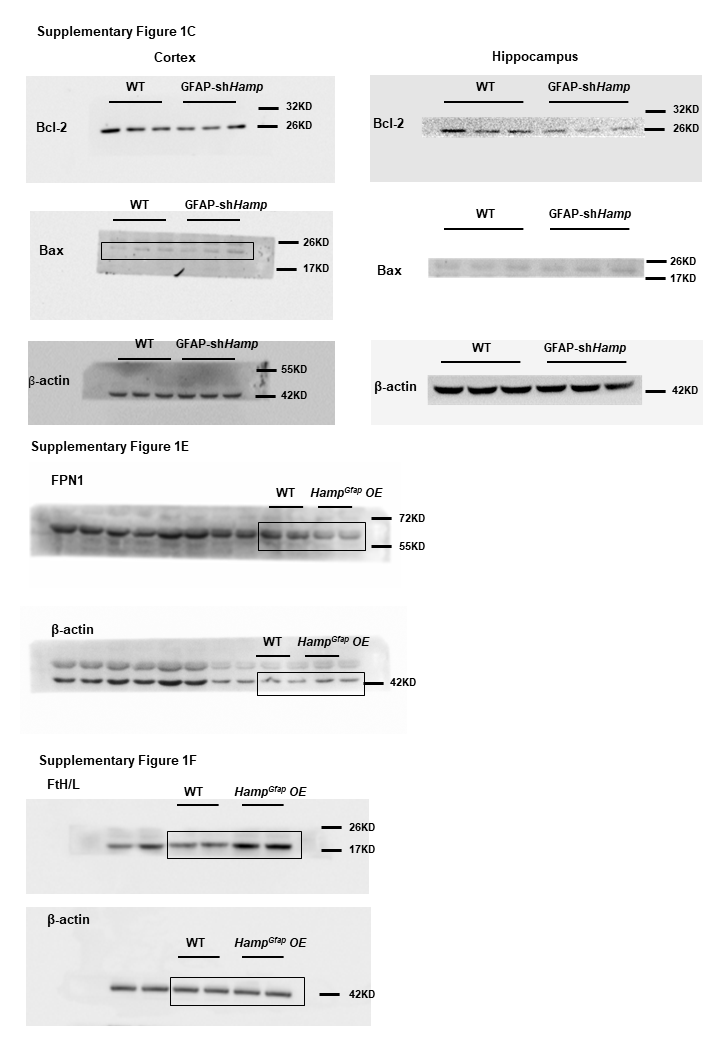

Supplement: Supplementary file 8 — Original Data File [file 41419_2022_5043_MOESM8_ESM.tif]

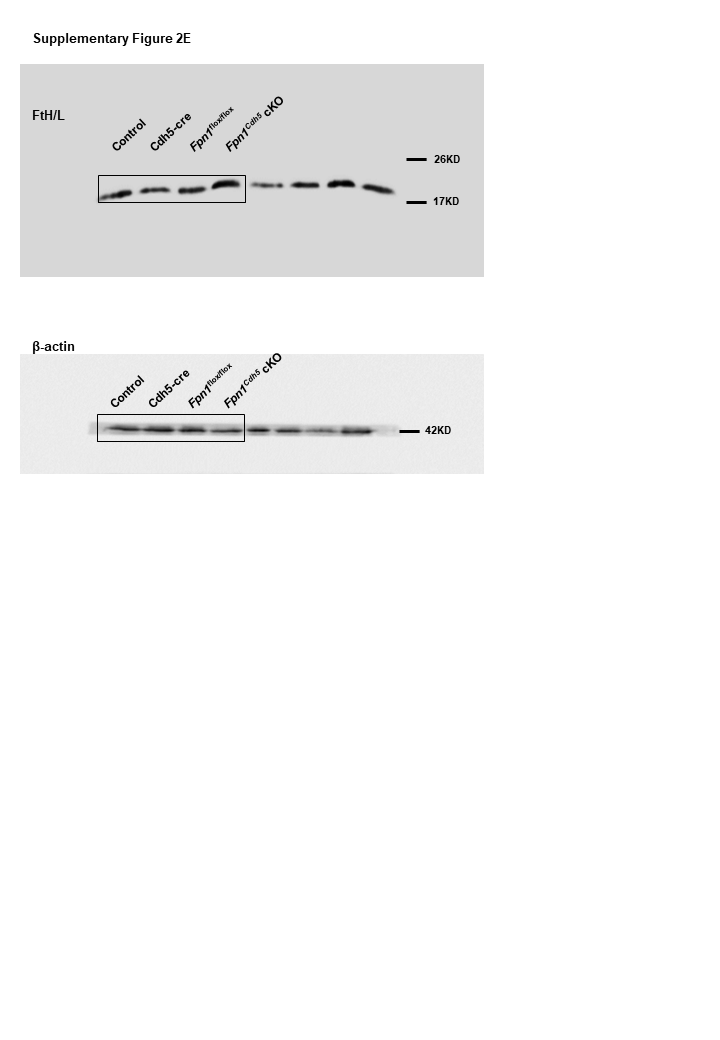

Supplement: Supplementary file 9 — Original Data File [file 41419_2022_5043_MOESM9_ESM.tif]
